# Genetic Burden of *TNNI3K* in Diagnostic Testing of Patients With Dilated Cardiomyopathy and Supraventricular Arrhythmias

**DOI:** 10.1161/CIRCGEN.122.003975

**Published:** 2023-05-18

**Authors:** Caroline Pham, Karolina Andrzejczyk, Sean J. Jurgens, Ronald Lekanne Deprez, Kaylin C.A. Palm, Alexa M.C. Vermeer, Janneke Nijman, Imke Christiaans, Daniela Q.C.M. Barge-Schaapveld, Pascal F.H.M. van Dessel, Leander Beekman, Seung Hoan Choi, Steven A. Lubitz, Doris Skoric-Milosavljevic, Lisa van den Bersselaar, Philip R. Jansen, Jaël S. Copier, Patrick T. Ellinor, Arthur A.M. Wilde, Connie R. Bezzina, Elisabeth M. Lodder

**Affiliations:** 1 Department of Experimental Cardiology (C.P., K.A., S.J.J., K.C.A.P., L.B., J.S.C., C.R.B., E.M.L.), Heart Center, Amsterdam UMC location University of Amsterdam, the Netherlands.; 2 Department of Cardiology (A.A.M.W.), Heart Center, Amsterdam UMC location University of Amsterdam, the Netherlands.; 3 Amsterdam Cardiovascular Sciences, Heart Failure and Arrhythmias, the Netherlands (C.P., K.A., S.J.J., K.C.A.P., L.B., J.S.C., A.A.M.W., C.R.B., E.M.L.).; 4 Cardiovascular Disease Initiative, Broad Institute of MIT and Harvard, Cambridge, MA (S.J.J., S.A.L., P.T.E.).; 5 Cardiovascular Research Center, Massachusetts General Hospital, Boston (S.J.J., S.A.L., P.T.E.).; 6 Department of Human Genetics, Amsterdam UMC location University of Amsterdam, the Netherlands (R.L.D., A.M.C.V., J.N., D.S.-M., P.R.J., E.M.L.).; 7 Department of Genetics, University Medical Center Groningen, University of Groningen, the Netherlands (I.C.).; 8 Department of Cardiology, Thorax Center Twente, Medisch Spectrum Twente (MST), Enschede, the Netherlands (P.F.H.M.v.D.).; 9 Department of Clinical Genetics, Leiden University Medical Center, Leiden, The Netherlands (D.Q.C.M.B.-S.).; 10 Department of Biostatistics, Boston University, MA (S.H.C.).; 11 Amsterdam UMC location Vrije Universiteit Amsterdam, Department of Complex Trait Genetics, the Netherlands (P.R.J.).; 12 Department of Clinical Genetics, Erasmus MC, University Medical Center, Rotterdam, the Netherlands (L.v.d.B.).

**Keywords:** cardiac arrhythmias, dilated cardiomyopathy, genetics, phosphorylation

## Abstract

**BACKGROUND:**

Genetic variants in *TNNI3K* (troponin-I interacting kinase) have previously been associated with dilated cardiomyopathy (DCM), cardiac conduction disease, and supraventricular tachycardias. However, the link between *TNNI3K* variants and these cardiac phenotypes shows a lack of consensus concerning phenotype and protein function.

**METHODS:**

We describe a systematic retrospective study of a cohort of patients undergoing genetic testing for cardiac arrhythmias and cardiomyopathy including *TNNI3K*. We further performed burden testing of *TNNI3K* in the UK Biobank. For 2 novel *TNNI3K* variants, we tested cosegregation. TNNI3K kinase function was estimated by TNNI3K autophosphorylation assays.

**RESULTS:**

We demonstrate enrichment of rare coding *TNNI3K* variants in DCM patients in the Amsterdam cohort. In the UK Biobank, we observed an association between *TNNI3K* missense (but not loss-of-function) variants and DCM and atrial fibrillation. Furthermore, we demonstrate genetic segregation for 2 rare variants, TNNI3K-p.Ile512Thr and TNNI3K-p.His592Tyr, with phenotypes consisting of DCM, cardiac conduction disease, and supraventricular tachycardia, together with increased autophosphorylation. In contrast, TNNI3K-p.Arg556_Asn590del, a likely benign variant, demonstrated depleted autophosphorylation.

**CONCLUSIONS:**

Our findings demonstrate an increased burden of rare coding *TNNI3K* variants in cardiac patients with DCM. Furthermore, we present 2 novel likely pathogenic *TNNI3K* variants with increased autophosphorylation, suggesting that enhanced autophosphorylation is likely to drive pathogenicity.

**See Editorial by Rieder et al**

Up to now, 7 *TNNI3K* (troponin-I interacting kinase) variants have been reported,^1–7^ from which 3 with moderate-to-strong genetic evidence, for example, multigenerational cosegregation.^1,2,4^ Patients harboring variants in *TNNI3K* present with several cardiac phenotypes including dilated cardiomyopathy (DCM), cardiac conduction disease (CCD), and supraventricular tachycardias (SVT). However, the evidence linking *TNNI3K* to human cardiac disease has thus far remained limited to family reports, some with limited genetic evidence.^1–7^

TNNI3K is a dual-specific (tyrosine and serine/threonine) kinase, which is mainly expressed in the heart and is conserved between species.^8,9^ Unfortunately, the phosphorylation targets of TNNI3K are still largely unidentified. However, previous work in murine models indicates that increased TNNI3K levels or activity are associated with cardiac conduction delay,^10^ exacerbation of cardiomyopathy,^9,11,12^ and reduced repair after ischemia reperfusion injury.^13–15^ Previous in vitro testing of TNNI3K autophosphorylation of 3 likely pathogenic human variants demonstrated an enhanced kinase function for TNNI3K-p.Glu768Lys,^4^ whereas p.Gly526Asp^1^ and p.Thr539Ala^2^ showed decreased autophosphorylation.^4^ While loss-of-function (LoF) variants in *TNNI3K* have been described,^1,2^ the pathogenicity of such variants is debated due to the lack of genetic evidence and the presence of homozygous LoF variants in the general population.^15^

Despite previous reports in families and extensive work in mice, many questions remain about the effects of genetic variations in *TNNI3K* on cardiac disease and the direction of effects. Here, we show the yield of 3 years of diagnostic testing of *TNNI3K* in arrhythmia and cardiomyopathy patients. In addition, we compared the burden in *TNNI3K* variants in individuals referred for cardiac genetic testing with the prevalence in gnomAD exomes.^16^ This burden test was replicated in the UK Biobank^17^ as external validation. Furthermore, we performed functional analyses of in vitro kinase activity for variants with the most data for potential cosegregation, a novel published variant,^6^ and a common variant in the Dutch population.

## METHODS

The authors declare that all supporting data are available within the article. Clinical and genetic studies were approved by the Medical Ethics Review Committee. Informed consent was obtained from all individuals of which the clinical data were described. Patients provided general consent for DNA studies in the setting of the Amsterdam UMC Cardiogenetics biobank (BTC 2014-003 A201435 and VUmc_2020_4231). Full methods are available as Supplemental Material.

## RESULTS

### The Outcome of Genetic Screening of *TNNI3K*

We identified 52 probands harboring 36 unique rare variants in *TNNI3K* matching the inclusion criteria in a total of 2467 tested individuals. A complete overview of all identified probands and the associated variants and phenotypes is given in Table S6. In addition to the rare variants in *TNNI3K*, probands carried rare variants in other screened genes (Table S6), most were class 3 missense variants in *TTN*, and 2 (likely) pathogenic variants in *TTN*. Patients carrying these additional variants did not present with a different phenotype, indicating that these variants are likely benign.

In addition to the rare coding variants described earlier, we followed up on a variant with a minor allele frequency (MAF) of 0.0018, that is, above our filtering criteria (rs145260115) as the first patient with this variant was a homozygous carrier. This variant, c.1772G>C, is predicted to result in p.Ser591Thr. As the variant is located near a splice junction, we performed in vitro splicing analysis, which revealed that the variant leads to a loss of splice donor/acceptor pair causing an in-frame deletion: p.Arg556_Asn590del (Figure S2). In total, 19 carriers of this variant were identified, including 1 homozygous individual. One of the heterozygous carriers was also carrying another variant in *TNNI3K*.

### Enrichment Analysis

**Figure 1.**
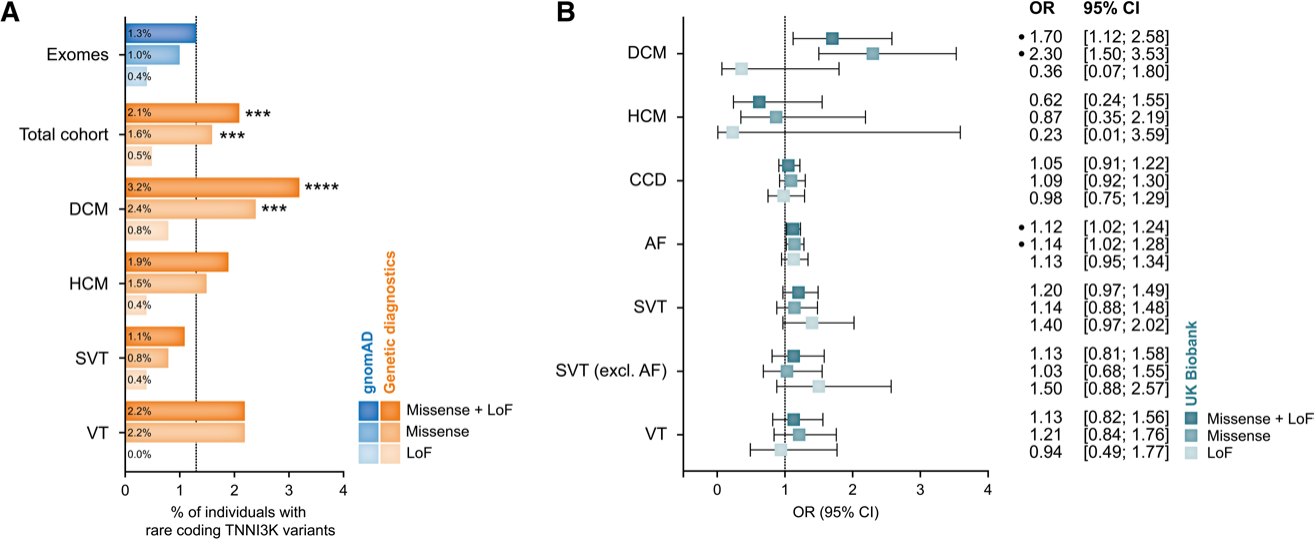
**Enrichment analysis of rare coding variants in *TNNI3K* in patients with cardiac disease. A**, Percentages of individuals with rare coding *TNNI3K* variants in gnomAD exomes (blue) vs genetic diagnostics (orange). The χ^2^ test vs gnomAD exomes: ***P<0.001 and ****P<0.0001. **B**, Odds ratio (OR) and 95% CIs of rare coding *TNNI3K* variants among cardiac traits in the UK Biobank. Closed circles indicate a significant association between the cardiac trait and *TNNI3K* variants. AF indicates atrial fibrillation; CCD, cardiac conduction disease; DCM, dilated cardiomyopathy; HCM, hypertrophic cardiomyopathy; LoF, loss-of-function; SVT, supraventricular tachycardia; and VT, ventricular tachycardia.

In the studied period, 2467 probands underwent genetic examination of the arrhythmia or cardiomyopathy panel including *TNNI3K*, or both. In this group, 52 individuals carried a rare (MAF <0.0001) coding variant in *TNNI3K* (2.1%). We compared the study group with the gnomAD exomes v2.1 consisting of the genetic data of 125 748 individuals, which is comparable to the general population with a similar burden of cardiovascular disease.^16,18^ After filtering for MAF and protein-altering changes, 1649 rare *TNNI3K* alleles were identified in this cohort (1.3%). This constitutes a 1.6-fold overrepresentation of rare *TNNI3K* variants (2.1%) in our study cohort compared with gnomAD (Figure 1A). We further investigated the reasons for referral for genetic testing with a focus on DCM, as the major phenotype previously associated with variants in *TNNI3K*. In the total study cohort, 622 individuals were referred with a main diagnosis of DCM, of these 20 (3.2%, 2.5-fold more than in gnomAD exomes) carried a variant in *TNNI3K*. Other indications for referral included the following: hypertrophic cardiomyopathy (1.9% *TNNI3K* variant carriers in the total cohort), SVT (1.1% *TNNI3K* carriers variant in the total cohort), and ventricular tachycardia (2.2% *TNNI3K* variant carriers in the total cohort).

We next sought to determine whether *TNNI3K* variants contribute to cardiac diseases in the general population, without the inclusion bias inherent to a cohort referred for genetic testing. To this end, we turned to the UK Biobank, a population-based dataset with rich phenotypic data and exome sequencing on over 450 000 individuals.^19^ We performed gene-based burden testing of rare *TNNI3K* variants for 7 disease end points^20^ (atrial fibrillation [AF], SVT, SVT [excl. AF], DCM, hypertrophic cardiomyopathy, CCD, and ventricular tachycardia) across 454 162 individuals. We performed burden testing over a range of rare variant masks, producing a single *P*-value per disease using the Cauchy distribution (Supplemental Methods; Tables S7 through S9). We found that rare variants in *TNNI3K* were significantly associated with DCM in this analysis (*P*=0.003; Figure 1B; Table S9). Consistent with the notion that LoF variants do not produce a cardiac phenotype, we found that this signal was entirely driven by missense variants (*P*=0.001; n_carriers_=4950; odds ratio [OR, 95% CI]=2.30 [1.5; 3.53]), while LoF variants were not associated with DCM (*P*=0.4; n_carriers_=1981; OR [95% CI]=0.36 [0.07; 1.8]). The power for detecting an effect of LoF variations in DCM is 80% to 100% for an OR of 2.8 to 3.9 (1-sided) or 3.1 to 4.3 (2-sided), respectively. This is in the order of magnitude we would expect for LoF variants should this be the underlying mechanism. The OR for the arguably more diluted signal in the missense variants is 2.3. We further identified an association with AF (*P*=0.03), which was again completely driven by missense (*P*=0.03; n_carriers_=5122; OR [95% CI]=1.14 [1.02; 1.28]) rather than LoF variants (*P*=0.2; n_carriers_=2046; OR [95% CI]=1.13 [0.95; 1.34]). In a sensitivity analysis focused on European ancestry, we found that the associations with DCM and AF were robust (*P*=0.006 and *P*=0.03, respectively). In aggregate, rare *TNNI3K* missense and LoF variants were identified in 2.6% of DCM cases, compared with 1.6% of controls (Table S10). These results mirror our clinical genetics cohort-based findings highlighting DCM, and associated SVT, as important phenotypic consequences of *TNNI3K* missense variations.

We further aimed to characterize the phenotypic outcome for complete loss of *TNNI3K* in humans. We identified 1 homozygous carrier of a rare *TNNI3K* LoF variant among the 454 162 individuals in the UK Biobank. This individual (in her sixties) was seemingly not affected by arrhythmia, heart failure, cardiomyopathy, or CCD.

### Clinical Presentation Among *TNNI3K* Variant Carriers

**Figure 2.**
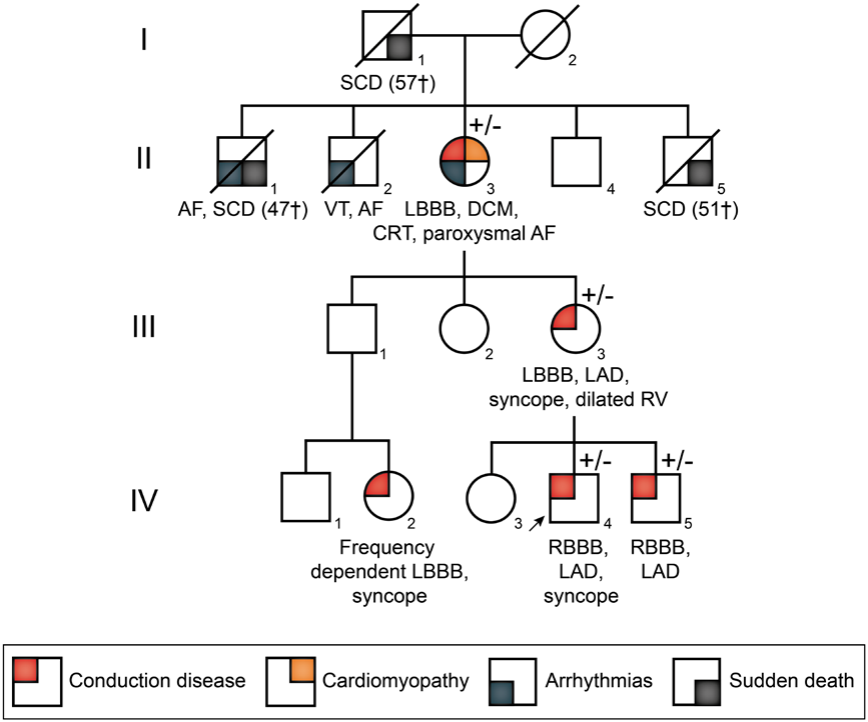
**Pedigree of a family carrying TNNI3K-p.Ile512Thr.** Arrow designates the proband. Heterozygous carriers of TNNI3K-p.Ile512Thr are specified by +/−. Diagonal lines indicate deceased individuals. The number in the diamond denotes the number of individuals. AF indicates atrial fibrillation; CRT, cardiac resynchronization therapy; DCM, dilated cardiomyopathy; LAD, left axis deviation; LBBB, left bundle branch block; RBBB, right bundle branch block; and SCD, sudden cardiac death.

We examined the available clinical data of all individuals and follow-up cascade screening in families with multiple-affected individuals. From these, we identified 2 rare variants in TNNI3K (p.Ile512Thr and p.His592Tyr) for which sufficient data was provided to suggest segregation with the cardiac phenotype. TNNI3K-p.Ile512Thr was identified in 1 multigenerational family, including 4 heterozygous carriers and multiple-affected individuals unavailable for genetic screening (Figure 2). The clinical picture in this family consisted of multiple cases of sudden cardiac death at a young age, age-dependent DCM, and conduction disorders including right or left bundle branch blocks and left axis deviation (Figure 2)

**Figure 3.**
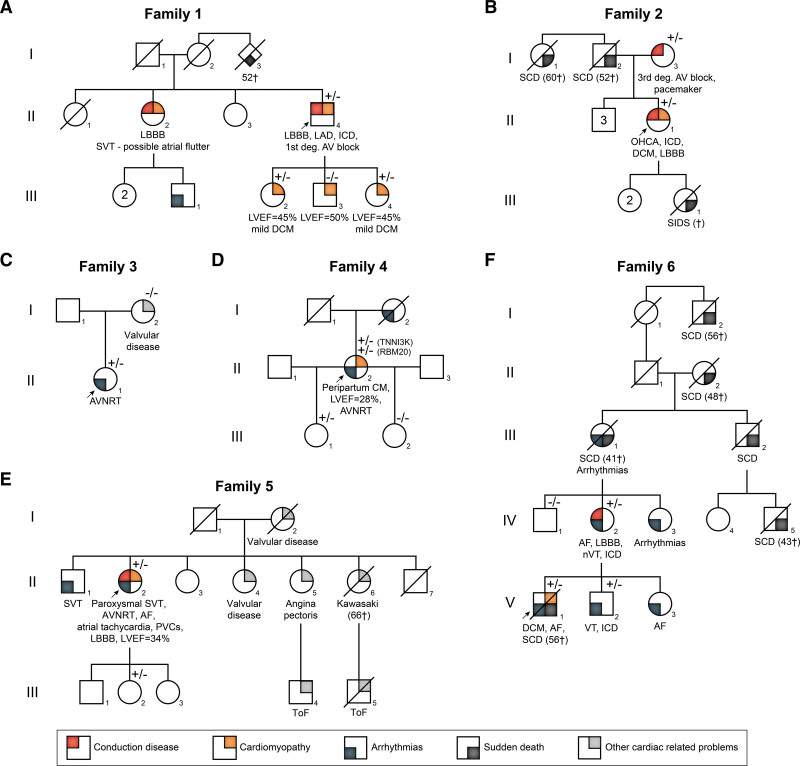
**Pedigrees of six families carrying TNNI3K-p.His592Tyr. A–F**, Arrows designate the probands. Heterozygous carriers of TNNI3K-p.His592Tyr are specified by +/−. Diagonal lines indicate deceased individuals. The number in the diamond denotes the number of individuals. AF indicates atrial fibrillation; AV, atrioventricular; AVNRT, AV-nodal reentry tachycardia; CRT, cardiac resynchronization therapy; DCM, dilated cardiomyopathy; LAD, Left axis deviation; LBBB, left bundle branch block; LVEF, left ventricular ejection fraction; nVT, nonsustained ventricular tachycardia; OHCA, out-of-hospital cardiac arrest; RBBB, right bundle branch block; SCD, sudden cardiac death; SIDS, sudden infant death syndrome; SVT, supraventricular tachycardia; ToF, tetralogy of Fallot; and VT, ventricular tachycardia.

In total, 13 heterozygous carriers of TNNI3K-p.His592Tyr were identified across 7 families (including 1 family known in diagnostics only based on the consent). The 6 families with complete informed consent are depicted in Figure 3. The clinical presentation of the carriers consisted of a combined phenotype of DCM (4/13), CCD (5/13), SVT (5/13; including atrioventricular-nodal reentry tachycardia, AF, atrial tachycardia, and possible junctional tachycardia), and ventricular tachycardia/sudden cardiac death (3/13). An additional 9 potential carriers in the families died suddenly at young age ≤60 years. However, not all TNNI3K-p.His592Tyr variant carriers exhibit a cardiac phenotype (4.III-1 [30 y/o] and 5.III-2 [41 y/o]). We suspect an age-dependent onset of cardiac traits resulting from TNNI3K-p.His592Tyr variant; hence, the missing cardiac phenotype in the 2 young carriers. The full overview of the clinical characteristics of both variants is shown in Supplemental Results; Table S11; Figure 3.

The combined annotation-dependent depletion score for in silico prediction of variant pathogenicity (GRCh38-v1.6) equals 24.9 and 25.4 (score ≥20 indicates the 1% most deleterious variants based on the prediction) for p.Ile512Thr and p.His592Tyr, respectively.^21^ Both variants were absent from gnomAD v2.1.1 (transcript ENST00000326637.3). The highest MAF for p.His592Tyr and p.Ile512Thr was <0.00004 and 0, respectively, based on the gnomAD v3.1.2 (transcript ENST00000326637.8).

### Autophosphorylation of Detected TNNI3K Variants

**Figure 4.**
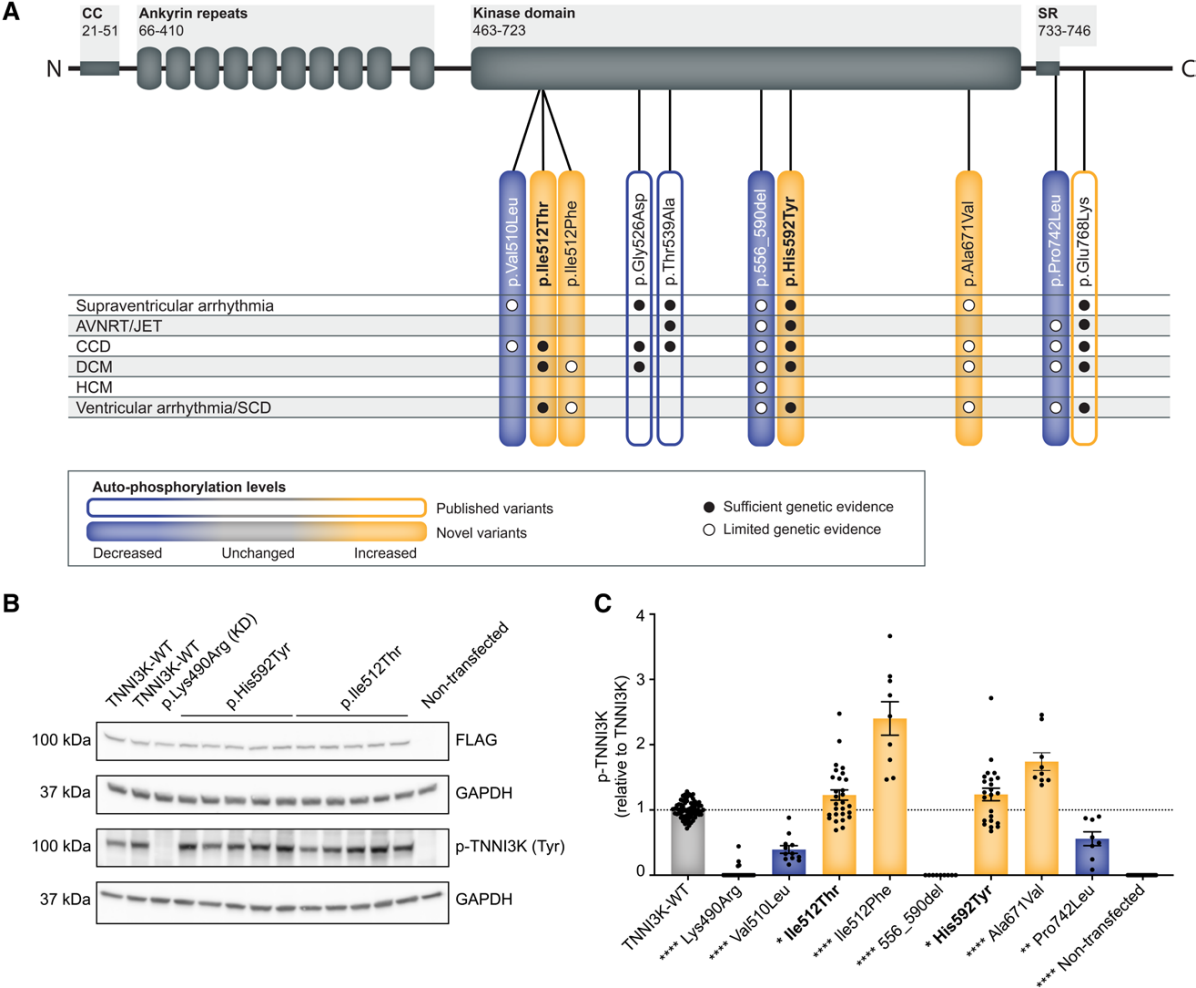
**Autophosphorylation assay of TNNI3K variants. A**, Schematic illustration depicting the TNNI3K protein and the location of the variants. Numbers represent the amino acid position. **B**, Western blot of HEK293A cell lysates transfected with FLAG-tagged TNNI3K, TNNI3K-p.Lys490Arg (KD, kinase-dead control), TNNI3K-p.His592Tyr, or TNNI3K-pIle512Thr. Nontransfected cell lysates were included as a negative control. TNNI3K expression was indicated with an anti-FLAG antibody and phosphorylated TNNI3K (p-TNNI3K) was detected with an anti-phospho-tyrosine antibody. GAPDH was included as a loading control. **C**, Western blot analysis of TNNI3K variants. Each dot represents an independent transfection. p-TNNI3K expression was corrected for FLAG expression. Values are relative to TNNI3K expression. Data are shown as mean±SEM. One-way ANOVA (Dunnett’s) or Kruskal-Wallis (Dunn’s) vs TNNI3K *P<0.05, **P<0.01, ***P<0.001, ****P<0.0001 (indicated on the *x*-axis). ANK indicates ankyrin repeat; AVNRT, AV-nodal reentry tachycardia; CC, coiled-coil domain; CCD, cardiac conduction disease; DCM, dilated cardiomyopathy; HCM, hypertrophic cardiomyopathy; JET, junctional ectopic tachycardia; SCD, sudden cardiac death; and SR, serine-rich domain.

To test the TNNI3K kinase function, we performed an in vitro autophosphorylation assay for (I) TNNI3K-c.1774 C>T; p.(His592Tyr) and c.1535T>C; p.(Ile512Thr), (II) 10 of the most promising variants identified, and (III) for the recently reported recessive TNNI3K-p.Ser511Pro variant.^6^ Furthermore, we tested the autophosphorylation of rs145260115 (*TNNI3K*-c.1772G>C; r.1668_1772del): p.Arg556_Asn590del (see RNA analysis, Figure S2; Figure 4A).

Similar autophosphorylation levels were observed in the independent preparations of wild-type TNNI3K (Figure 4B and 4C; Figure S4: noncropped membrane). The negative controls TNNI3K-kinase-dead (p.Lys490Arg) variant and the nontransfected HEK293A cell lysate did not show any phosphorylation signal at the height of TNNI3K. Similar to the pathogenic TNNI3K-p.Glu768Lys described by Podliesna et al,^4^ both the p.His592Tyr and p.Ile512Thr variants demonstrated increased autophosphorylation levels of TNNI3K compared with TNNI3K-wild-type, indicating an enhanced kinase function. Increased TNNI3K phosphorylation was further detected for the p.Ile512Phe and p.Ala671Val variants. On the contrary, decreased TNNI3K autophosphorylation was observed in p.Val510Leu and p.Pro742Leu. The commonly observed (rs145260115) *TNNI3K*-c.1772G>C variant, which creates an alternative splice site resulting in an in-frame deletion of 34 amino acids (p.Arg556_Asn590del), demonstrated a total loss of kinase activity. The predicted p.Ser591Thr (which was not observed on cDNA level) does not affect autophosphorylation. The previously described TNNI3K-p.Ser511Pro variant^6^ did not show altered autophosphorylation levels (Figure S3). A complete overview of all kinase assays and the summarized outcome can be found in Figure S3; Figure 4.

## DISCUSSION

### Increased Burden of *TNNI3K* Variants in Cardiac Patients

Thus far the published literature concerning the link between genetic variants in *TNNI3K* and cardiac phenotypes shows a clear lack of consensus concerning phenotype and direction of effect.^15^ We here describe a systematic retrospective study of a cohort of patients undergoing genetic testing for cardiac arrhythmias and cardiomyopathy. Out of the 2467 screened patients, 52 (2.1%) were found to carry a rare (MAF<0.0001) and protein-changing variant in *TNNI3K*. This constitutes a 1.6-fold overrepresentation in comparison with the gnomAD exomes reference cohort.^16^ This observed burden is 2.5-fold stronger in patients with a primary diagnosis of DCM as an indication for genetic testing. This increased burden of rare coding variants in *TNNI3K* in patients with DCM was independently replicated in the UK Biobank for missense variants (2.5% versus 1.1%). As CCD, SVT, and AF are generally no indications for genetic testing, insufficient numbers of patients were present in our cohort to test for burden for these phenotypes. However, in the UK Biobank, a significant increase in carriers of *TNNI3K* variants was observed for DCM and AF compared with controls. No increased burden was found for LoF variants in *TNNI3K*, indicating that the observed total burden effect is driven by missense variants in *TNNI3K*.

### The Clinical Presentation of *TNNI3K* Variant Carriers

To date, genetic variants in *TNNI3K* have been associated with a mixed clinical picture including DCM, CCD, and (supra)ventricular arrhythmias.^1,2,4^ In this cohort, most variants in *TNNI3K* were identified in patients with limited available family history, precluding further cosegregation; which were therefore classified as variants of unknown significance (class 3).^22^ Based on the increased burden of such variants in our patients, it is likely that a part of these variants contributes to the phenotype of the carriers, especially of those with DCM, CCD, or SVT. As expected in many of these patients, additional variants of unknown significance were identified in other genes in the panels. These consisted largely of missense variants in *TTN* and 2 (likely) pathogenic variants in *TTN* in line with the known complex genetic architecture of *TTN*.^23^

For 2 variants, TNNI3K-p.Ile512Thr and TNNI3K-p.His592Tyr, we were able to perform cosegregation analysis in 1 and multiple pedigrees, respectively. Both variants showed strong genetic segregation with the phenotype consisting of DCM, CCD, and SVT, in line with the previously described pathogenic variants.^1,2,4^ In particular, the TNNI3K-p.His592Tyr variant (now reclassified as a class 4 likely pathogenic variant) was identified in 7 independent probands. The clinical history of these probands and their family members revealed the cooccurrence of age-dependent structural abnormalities and conduction disturbances, including atrioventricular blocks, bundle branch blocks, and supraventricular and junctional arrhythmias. In TNNI3K-p.His592Tyr variant carriers, we observed an increased burden of SVT (including atrioventricular-nodal reentry tachycardia, AF, atrial tachycardia, and possible junctional tachycardia) of 38% compared with an incidence of ~0.25% in the general population.^24^ The fact that we do not identify enrichment for SVT in the UK biobank data could be due to atrioventricular-nodal reentry tachycardia/SVT being specific to the p.His592Tyr variant. This is, however, unlikely considering the published TNNI3K-p.Glu768Lys families where a similar phenotype was observed.^4^ Another potential cause could be that a potential signal for atrioventricular-nodal reentry tachycardia in *TNNI3K* variant carriers was lost in the more broad SVT phenotype obtained from the UK Biobank.

At the time of admission, patients usually demonstrate mixed phenotypes with DCM and coexisting arrhythmias. As DCM can be the cause of arrhythmias, but also the consequence, it is difficult to determine the predominance of the structural or functional component in the development of full clinical manifestation.^25^ Further investigation and follow-up of all the families are currently ongoing.

### The Direction of Effect of *TNNI3K* Variants

The 3 previously published autosomal dominant variants in *TNNI3K*, with segregation data, provide contradicting evidence concerning the role of the kinase function of TNNI3K.^1,2,4^ In vitro autophosphorylation indicated a loss of kinase function for p.Gly526Asp, p.Thr539Ala^1,2^ and an increased autophosphorylation for the TNNI3K-p.Glu768Lys variant.^4^ Nevertheless, the clinical phenotype of genetic variation carriers strongly overlaps for all 3 variants. In line with TNNI3K-p.Glu768Lys, both the TNNI3K-p.Ile512Thr and TNNI3K-p.His592Tyr presented in this study show an increased level of autophosphorylation. Of note, also the p.Ile512Phe variant (ie, at the same position as the p.Ile512Thr variant) identified in 2 independent probands with DCM and ventricular tachycardia demonstrated increased TNNI3K autophosphorylation in vitro.

Next to the rare variants in *TNNI3K*, we detected rs145260115 (*TNNI3K*-c.1772G>C) in 17 individuals in our cohort. This variant has a population frequency of 0.2% in gnomAD^16^ and almost 2% (9/499 individuals) in the Dutch GoNL reference cohort.^26^ This variant was originally found in a homozygous carrier in our cohort and therefore investigated further, despite the relatively high frequency in the population. PCR analysis of blood-derived cDNA of the carriers revealed that rs145260115: c.1772G>C creates an abnormal splice donor site resulting in an in-frame deletion of 105 nucleotides (r.1668_1772del), leading to a deletion of 34 amino acids (p.Arg556_Asn590del) instead of the predicted missense variant p.Ser591Thr. The in vitro autophosphorylation assay indicates that this deletion leads to a complete loss of the kinase activity. Considering the proportion of the population carrying this variant: 5 and 50 times more prevalent than DCM for gnomAD and GoNL, respectively,^16,26,27^ this result suggests that a heterozygous loss of kinase function is likely not pathogenic. Therefore, the underlying molecular mechanism for the p.Gly526Asp and p.Thr539Ala variants remains to be solved. The interpretation of recessive LoF variants and thus the homozygous carrier in our cohort is still unsolved due to a lack of data. The Hardy-Weinberg prediction for homozygosity of rs145260115 (0.03% of the population) based on the heterozygous GoNL carriers is still relatively high in comparison to the prevalence of DCM. Notably, homozygous LoF variants in *TNNI3K* are observed in both gnomAD and the UK Biobank. In the UK Biobank, at least 1 homozygous LoF carrier did not have ICD code diagnoses for the studied cardiac diseases at the age of 63 years. These findings, in combination with the lack of burden of LoF variants in *TNNI3K* in patients with DCM, suggest that LoF variants may not be pathogenic, although this will require further study given currently the small numbers of homozygous carriers.

In summary, 3 years of diagnostic testing of *TNNI3K* in arrhythmia and cardiomyopathy patients yielded 2 missense variants (TNNI3K-p.Ile512Thr [1 family] and TNNI3K-p.His592Tyr [7 families]) in *TNNI3K* with cosegregation with DCM, SVT, and CCD. The latter variant is potentially a founder variant in the Netherlands. We further demonstrate the enrichment of rare coding *TNNI3K* variants in individuals with a cardiac burden, in particular DCM. This burden was independently replicated in the UK Biobank for DCM and AF. Functional studies revealed an increased kinase capacity for both likely pathogenic variants and a complete loss of kinase function in a variant with a population frequency higher than the associated phenotypes. Therefore, we suggest that enhanced TNNI3K kinase function is more likely to be pathogenic than when the kinase function is diminished. This is in line with previous data collected from TNNI3K-kinase-dead mouse models.^15^ However, further studies are required to decipher the molecular mechanism of TNNI3K and clarify its role in cardiac diseases in particular for homozygous LoF variants.

## ARTICLE INFORMATION

### Sources of Funding

This research was funded by the Netherlands CardioVascular Research Initiative: CVON2017-15 RESCUED, the Dutch Research Council: NWO Talent Scheme VIDI-91718361 (Drs Lodder, K. Andrzejczyk, and C. Pham), and the AMC funding scheme (J.S. Copier and Dr Lodder). S.J. Jurgens is supported by a Junior Clinical Scientist Fellowship (03-007-2022-0035) from the Dutch Heart Foundation, and by an Amsterdam UMC Doctoral Fellowship (Hartstichting). Dr Ellinor is supported by grants from the National Institutes of Health (1RO1HL092577, 1R01HL157635, 1R01HL157635), by a grant from the American Heart Association Strategically Focused Research Networks (18SFRN34110082), and by a grant from the European Union (MAESTRIA 965286). Dr Lubitz is a full-time employee of Novartis as of July 18, 2022. Prior to his employment at Novartis and during this work, he was supported by NIH grants R01HL139731, R01HL157635 and American Heart Association 18SFRN34250007.

### Disclosures

Dr Ellinor receives sponsored research support from Bayer AG and IBM Research; he has also served on advisory boards or consulted for Bayer AG, MyoKardia and Novartis. Dr Lubitz has received sponsored research support from Bristol Myers Squibb, Pfizer, Boehringer Ingelheim, Fitbit, Medtronic, Premier, and IBM, and has consulted for Bristol Myers Squibb, Pfizer, Blackstone Life Sciences, and Invitae. The other authors report no conflicts.

### Supplemental Material

Supplemental Methods

Tables S1–S11

Figures S1–S4

Supplemental Results

References^28–37^

## Supplementary Material
