# Supplementary material for: Genetic Burden of TNNI3K in Diagnostic Testing of Patients With Dilated Cardiomyopathy and Supraventricular Arrhythmias
Source: Circ Genom Precis Med. 2023 May 18;16(4):328–36. doi: 10.1161/CIRCGEN.122.003975 (PMC10426786; doi:10.1161/CIRCGEN.122.003975)
Supplement: Supplementary file 1 [file hcg-16-328-s001.pdf]

SUPPLEMENTAL MATERIAL

Supplemental Tables

Table I. Genes included in the cardiomyopathy and arrhythmia genetic screening panels from 2018 to 2022

**Cardiomyopathy**					
V18			V19		
1	*ACTC1*	(NM_005159.4)	1	*ACTC1*	(NM_005159.4)
2	*ACTN2*	(NM_001103.2; NM_001278343.1; NM_001278344.1)	2	*ACTN2*	(NM_001103.2; NM_001278343.1; NM_001278344.1)
3	*ALPK3*	(NM_020778.4)	3	*ALPK3*	(NM_020778.4)
4	*ANKRD1*	(NM_014391.2)	4	*ANKRD1*	(NM_014391.2)
5	*BAG3*	(NM_004281.3)	5	*BAG3*	(NM_004281.3)
6	*CALR3*	(NM_145046.3)	6	*CALR3*	(NM_145046.3)
7	*CAV3*	(NM_033337.2)	7	*CAV3*	(NM_033337.2)
8	*CDH2*	(NM_001792.4; NM_001308176.1)	8	*CDH2*	(NM_001792.4; NM_001308176.1)
9	*CRYAB*	(NM_001885.2)	9	*CRYAB*	(NM_001885.2)
10	*CSRP3*	(NM_003476.4)	10	*CSRP3*	(NM_003476.4)
11	*CTNNA3*	(NM_013266.3; NM_001291133.1)	11	*CTNNA3*	(NM_013266.3; NM_001291133.1)
12	*DES*	(NM_001927.3)	12	*DES*	(NM_001927.3)
13	*DSC2*	(NM_024422.4; NM_004949.4)	13	*DSC2*	(NM_024422.4; NM_004949.4)
14	*DSG2*	(NM_001943.4)	14	*DSG2*	(NM_001943.4)
15	*DSP*	(NM_004415.3)	15	*DSP*	(NM_004415.3)
16	*EMD (STA)*	(NM_000117.2)	16	*EMD (STA)*	(NM_000117.2)
17	*FHL1*	(NM_001159702.2; NM_001159701.1; NM_001159699.1)	17	*FHL1*	(NM_001159702.2; NM_001159701.1; NM_001159699.1)
18	*FHL2*	(NM_201555.1)	18	*FHL2*	(NM_201555.1)
19	*FKRP*	(NM_024301.4)	19	*FKRP*	(NM_024301.4)
20	*FLNC*	(NM_001458.4)	20	*FLNC*	(NM_001458.4)
21	*GLA*	(NM_000169.2)	21	*GLA*	(NM_000169.2)
22	*HCN4*	(NM_005477.2)	22	*HCN4*	(NM_005477.2)
23	*JPH2*	(NM_020433.4; NM_175913.3)	23	*JPH2*	(NM_020433.4; NM_175913.3)
24	*JUP*	(NM_021991.2)	24	*JUP*	(NM_021991.2)
25	*LAMA4*	(NM_001105206.2; NM_001105208.2)	25	*LAMA4*	(NM_001105206.2; NM_001105208.2)
26	*LAMP2*	(NM_002294.2; NM_013995.2; NM_001122606.1)	26	*LAMP2*	(NM_002294.2; NM_013995.2; NM_001122606.1)
27	*LDB3*	(NM_007078.2; NM_001080116.1)	27	*LDB3*	(NM_007078.2; NM_001080116.1)
28	*LMNA*	(NM_170707.3; NM_001257374.2; NM_005572.3; NM_001282624.1)	28	*LMNA*	(NM_170707.3; NM_001257374.2; NM_005572.3; NM_001282624.1)
29	*MIB1*	(NM_020774.2)	29	*MIB1*	(NM_020774.2)
30	*MYBPC3*	(NM_000256.3; intronic regions)	30	*MYBPC3*	(NM_000256.3; full introns)
31	*MYH6*	(NM_002471.3)	31	*MYH6*	(NM_002471.3)
32	*MYH7*	(NM_000257.3)	32	*MYH7*	(NM_000257.3)
33	*MYL2*	(NM_000432.3)	33	*MYL2*	(NM_000432.3)
34	*MYLK3*	(NM_182493.2)	34	*MYLK3*	(NM_182493.2)
35	*MYL3*	(NM_000258.2)	35	*MYL3*	(NM_000258.2)
36	*MYOZ2*	(NM_016599.4)	36	*MYOZ2*	(NM_016599.4)
37	*MYPN*	(NM_032578.2; NM_001256268.1)	37	*MYPN*	(NM_032578.2; NM_001256268.1)
38	*NEXN*	(NM_144573.3)	38	*NEXN*	(NM_144573.3)
39	*PKP2*	(NM_004572.3)	39	*PKP2*	(NM_004572.3)
40	*PLN*	(NM_002667.3)	40	*PLN*	(NM_002667.3)
41	*PPA2*	(NM_176869.2)	41	*PPA2*	(NM_176869.2)
42	*PRDM16*	(NM_022114.3)	42	*PRDM16*	(NM_022114.3)
43	*PRKAG2*	(NM_016203.3; NM_001304527.1)	43	*PRKAG2*	(NM_016203.3; NM_001304527.1)
44	*RBM20*	(NM_001134363.1)	44	*RBM20*	(NM_001134363.1)
45	*SCN5A*	(NM_198056.2)	45	*SCN5A*	(NM_198056.2)
46	*TAZ*	(NM_000116.3; NM_001303465.1)	46	*TAZ*	(NM_000116.3; NM_001303465.1)
47	*TCAP*	(NM_003673.3)	47	*TCAP*	(NM_003673.3)
48	*TMEM43*	(NM_024334.2)	48	*TMEM43*	(NM_024334.2)
49	*TNNC1*	(NM_003280.2)	49	*TNNC1*	(NM_003280.2)
50	*TNNI3*	(NM_000363.4)	50	*TNNI3*	(NM_000363.4)
51	*TNNI3K*	(NM_015978.2)	51	*TNNI3K*	(NM_015978.2)
52	*TNNT2*	(NM_000364.2; NM_001001430.2)	52	*TNNT2*	(NM_000364.2; NM_001001430.2)
53	*TPM1*	(NM_000366.5; NM_001018005.1; NM_001018020.1; NM_001301289.1)	53	*TPM1*	(NM_000366.5; NM_001018005.1; NM_001018020.1; NM_001301289.1)
54	*TTN*		54	*TTN*	
	N2-B	(NM_003319.4; all coding exones)		*N2-B*	(NM_003319.4; all coding exones)
	N2A	(NM_133378.4; all coding exones)		*N2A*	(NM_133378.4; all coding exones)
	Novex-3	(NM_133379.4; all coding exones)		*Novex-3*	(NM_133379.4; all coding exones)
	Novex-1	(NM_133432.3; all coding exones)		*Novex-1*	(NM_133432.3; all coding exones)
	Novex-2	(NM_133437.4; all coding exones)		*Novex-2*	(NM_133437.4; all coding exones)
	Transcript variant IC	(NM_001267550.2; 335 out of 362 coding exones)		*Transcript variant IC*	(NM_001267550.2; 335 out of 362 coding exones)
55	*TTR*	(NM_000371.3)	55	*TTR*	(NM_000371.3)
56	*VCL*	(NM_014000.2)	56	*VCL*	(NM_014000.2)
**Arrhythmia**					
V7			V8		
1	*ABCC9*	(NM_005691.3; NM_020297.2)	1	*ABCC9*	(NM_005691.3; NM_020297.2)
2	*AKAP9*	(NM_005751.4; NM_147185.2)	2	*AKAP9*	(NM_005751.4; NM_147185.2)
3	*ANK2*	(NM_001127493.2; NM_001148.5; NM_001354269.1; NM_001354230.1; NM_001354241.1; NM_001354274.1; NM_001354278.1)	3	*ANK2*	(NM_001127493.2; NM_001148.5; NM_001354269.1; NM_001354230.1; NM_001354241.1; NM_001354274.1; NM_001354278.1)
4	*ASPH*	(NM_020164.4)	4	*ASPH*	(NM_020164.4)
5	*CACNA1C*	(NM_000719.6; NM_001129827.1; NM_001129830.1; NM_001129840.1)	5	*CACNA1C*	(NM_000719.6; NM_001129827.1; NM_001129830.1; NM_001129840.1)
6	*CACNA1D*	(NM_000720.2; NM_001128839.2; NM_001128840.2)	6	*CACNA1D*	(NM_000720.2; NM_001128839.2; NM_001128840.2)
7	*CACNA2D1*	(NM_000722.2; NM_001302890.1)	7	*CACNA2D1*	(NM_000722.2; NM_001302890.1)
8	*CACNB2*	(NM_201596.2; NM_000724.3; NM_201570.2; NM_201572.3; NM_201590.2; NM_201593.2; NM_001167945.1;NM_001330060.1; NM_201597.2; NM_201571.3)	8	*CACNB2*	(NM_201596.2; NM_000724.3; NM_201570.2; NM_201572.3; NM_201590.2; NM_201593.2; NM_001167945.1;NM_001330060.1; NM_201597.2; NM_201571.3)
9	*CALM1*	(NM_006888.4)	9	*CALM1*	(NM_006888.4)
10	*CALM2*	(NM_001743.5; NM_001305624.1; NM_001305625.1)	10	*CALM2*	(NM_001743.5; NM_001305624.1; NM_001305625.1)
11	*CALM3*	(NM_005184.2; NM_001329921.1; NM_001329922.1)	11	*CALM3*	(NM_005184.2; NM_001329921.1; NM_001329922.1)
12	*CASQ2*	(NM_001232.3)	12	*CASQ2*	(NM_001232.3)
13	*CAV3*	(NM_033337.2)	13	*CAV3*	(NM_033337.2)
14	*DPP6*	(NM_001936 only position c.-340)	14	*DPP6*	(NM_001936 only position c.-340)
15	*GJA5*	(NM_005266.6; NM_181703.3)	15	*GJA5*	(NM_005266.6; NM_181703.3)
16	*GNB2*	(NM_005273.3)	16	*GNB2*	(NM_005273.3)
17	*GPD1L*	(NM_015141.3)	17	*GPD1L*	(NM_015141.3)
18	*HCN4*	(NM_005477.2)	18	*HCN4*	(NM_005477.2)
19	*JPH2*	(NM_020433.4; NM_175913.3)	19	*JPH2*	(NM_020433.4; NM_175913.3)
20	*KCNA5*	(NM_002234.3)	20	*KCNA5*	(NM_002234.3)
21	*KCND3*	(NM_004980.4)	21	*KCND3*	(NM_004980.4)
22	*KCNE1*	(NM_000219.5)	22	*KCNE1*	(NM_000219.5)
23	*KCNE5*	(NM_012282.3)	23	*KCNE5*	(NM_012282.3)
24	*KCNE2*	(NM_172201.1)	24	*KCNE2*	(NM_172201.1)
25	*KCNE3*	(NM_005472.4)	25	*KCNE3*	(NM_005472.4)
26	*KCNH2*	(NM_000238.3; NM_001204798.1; NM_172056.2)	26	*KCNH2*	(NM_000238.3; NM_001204798.1; NM_172056.2)
27	*KCNJ2*	(NM_000891.2)	27	*KCNJ2*	(NM_000891.2)
28	*KCNJ5*	(NM_000890.3)	28	*KCNJ5*	(NM_000890.3)
29	*KCNJ8*	(NM_004982.3)	29	*KCNJ8*	(NM_004982.3)
30	*KCNQ1*	(NM_000218.2; NM_181798.1)	30	*KCNQ1*	(NM_000218.2; NM_181798.1)
31	*LAMP2*	(NM_002294.2; NM_013995.2; NM_001122606.1)	31	*LAMP2*	(NM_002294.2; NM_013995.2; NM_001122606.1)
32	*LMNA*	(NM_170707.3; NM_001257374.2; NM_005572.3; NM_001282624.1)	32	*LMNA*	(NM_170707.3; NM_001257374.2; NM_005572.3; NM_001282624.1)
33	*MYL4*	(NM_002476.2)	33	*MYL4*	(NM_002476.2)
34	*NKX2-5*	(NM_004387.3; NM_001166176.1; NM_001166175.1)	34	*NKX2-5*	(NM_004387.3; NM_001166176.1; NM_001166175.1)
35	*NPPA*	(NM_006172.3)	35	*NPPA*	(NM_006172.3)
36	*PKP2*	(NM_004572.3)	36	*PKP2*	(NM_004572.3)
37	*PLN*	(NM_002667.4)	37	*PLN*	(NM_002667.4)
38	*PPA2*	(NM_176869.2)	38	*PPA2*	(NM_176869.2)
39	*PRKAG2*	(NM_016203.3; NM_001304527.1)	39	*PRKAG2*	(NM_016203.3; NM_001304527.1)
40	*RANGRF*	(NM_016492.4; NM_001177801.1; NM_001177802.1; NM_001330127.1)	40	*RANGRF*	(NM_016492.4; NM_001177801.1; NM_001177802.1; NM_001330127.1)
41	*RYR2*	(NM_001035.2)	41	*RYR2*	(NM_001035.2)
42	*SCN1B*	(NM_001037.4; NM_199037.4)	42	*SCN1B*	(NM_001037.4; NM_199037.4)
43	*SCN2B*	(NM_004588.4)	43	*SCN2B*	(NM_004588.4)
44	*SCN3B*	(NM_018400.3)	44	*SCN3B*	(NM_018400.3)
45	*SCN4B*	(NM_174934.3)	45	*SCN4B*	(NM_174934.3)
46	*SCN5A*	(NM_198056.2; NM_001160160.1)	46	*SCN5A*	(NM_198056.2; NM_001160160.1)
47	*SCN10A*	(NM_006514.2)	47	*SCN10A*	(NM_006514.2)
48	*SLMAP*	(NM_007159.4; NM_001304420.2; NM_001311178.1)	48	*SLMAP*	(NM_007159.4; NM_001304420.2; NM_001311178.1)
49	*SNTA1*	(NM_003098.2)	49	*SNTA1*	(NM_003098.2)
50	*TECRL*	(NM_001010874.4)	50	*TECRL*	(NM_001010874.4)
51	*TNNI3K*	(NM_015978.2)	51	*TNNI3K*	(NM_015978.2)
52	*TNNT2*	(NM_000364.3; NM_001001430.2)	52	*TNNT2*	(NM_000364.3; NM_001001430.2)
53	*TRDN*	(NM_006073.3)	53	*TRDN*	(NM_006073.3)
54	*TRPM4*	(NM_017636.3)	54	*TRPM4*	(NM_017636.3)
			55	*DPP6*	
			56	*HEY2*	
			57	*HOOK3*	
			58	*RRAD*	
			59	*TCAP*	
			60	*SLC4A3*	

Table II. Definitions for curated disease phenotypes in the UK Biobank

Traits	Field	Matching Code	Exclude
**Atrial fibrillation or flutter (AF)**	20002	1471;1483	NO
	20004	1524	NO
	40001	I48;I48.0;I48.1;I48.2;I48.3;I48.4;I48.9	NO
	40002	I48;I48.0;I48.1;I48.2;I48.3;I48.4;I48.9	NO
	41200	K57.1;K62.1;K62.2;K62.3;K62.4;X50.1;X50.2	NO
	41202	I48;I48.0;I48.1;I48.2;I48.3;I48.4;I48.9	NO
	41203	4273	NO
	41204	I48;I48.0;I48.1;I48.2;I48.3;I48.4;I48.9	NO
	41205	4273	NO
	41210	K57.1;K62.1;K62.2;K62.3;K62.4;X50.1;X50.2	NO
**Conduction system disease**	40001	I44;I44.0;I44.1;I44.2;I44.3;I44.4;I44.5;I44.6;I44.7;I45.0;I45.1;I45.2;I45.3;I45.4;I45.5	NO
	40002	I44;I44.0;I44.1;I44.2;I44.3;I44.4;I44.5;I44.6;I44.7;I45.0;I45.1;I45.2;I45.3;I45.4;I45.5	NO
	41202	I44;I44.0;I44.1;I44.2;I44.3;I44.4;I44.5;I44.6;I44.7;I45.0;I45.1;I45.2;I45.3;I45.4;I45.5	NO
	41203	4260;4261;4263;4264;4265;4266	NO
	41204	I44;I44.0;I44.1;I44.2;I44.3;I44.4;I44.5;I44.6;I44.7;I45.0;I45.1;I45.2;I45.3;I45.4;I45.5	NO
	41205	4260;4261;4263;4264;4265;4266	NO
**Dilated Cardiomyopathy**	41202	I42.0	NO
	41204	I42.0	NO
	40001	I42.0	NO
	40002	I42.0	NO
	20002	10.751.588	YES
	20004	107.010.951.523	YES
	40001	I25.5,I42.1,I42.2,Z95.1,Z95.5	YES
	40002	I25.5,I42.1,I42.2,Z95.1,Z95.5	YES
	41200	K40.1,K40.2,K40.3,K40.4,K40.8,K40.9,K41.1,K41.2,K41.3,K41.4,K41.8,K41.9,K44.1,K44.2,K44.8,K44.9,K45.1,K45.2,K45.3,K45.4,K45.5,K45.6,K45.8,K45.9,K46.1,K46.2,K46.3,K46.4,K46.5,K46.8,K46.9,K47.1,K49.1,K49.2,K49.3,K49.4,K49.8,K49.9,K50.1,K50.2,K50.4,K75.1,K75.2,K75.3,K75.4,K75.8,K75.9	YES
	41202	I25.5,I42.1,I42.2,Z95.1,Z95.5	YES
	41204	I25.5,I42.1,I42.2,Z95.1,Z95.5	YES
	41210	K40.1,K40.2,K40.3,K40.4,K40.8,K40.9,K41.1,K41.2,K41.3,K41.4,K41.8,K41.9,K44.1,K44.2,K44.8,K44.9,K45.1,K45.2,K45.3,K45.4,K45.5,K45.6,K45.8,K45.9,K46.1,K46.2,K46.3,K46.4,K46.5,K46.8,K46.9,K47.1,K49.1,K49.2,K49.3,K49.4,K49.8,K49.9,K50.1,K50.2,K50.4,K75.1,K75.2,K75.3,K75.4,K75.8,K75.9	YES
	42001	0,1,2	YES
**Hypertrophic Cardiomyopathy**	20002	1588	NO
	41202	I42.1,I42.2	NO
	41204	I42.1,I42.2	NO
	40001	I42.1,I42.2	NO
	40002	I42.1,I42.2	NO
	41203	4.251	NO
	41205	4251	NO
**Supraventricular tachycardia**	20002	1487	NO
	40001	I47.1	NO
	40002	I47.1	NO
	41200	K57.2;K57.5	NO
	41202	I47.1	NO
	41203	4270	NO
	41204	I47.1	NO
	41205	4270	NO
	41210	K57.2;K57.5	NO
**Supraventricular tachycardia excluding AF**	20002	1487	NO
	40001	I47.1	NO
	40002	I47.1	NO
	41200	K57.2;K57.5	NO
	41202	I47.1	NO
	41203	4270	NO
	41204	I47.1	NO
	41205	4270	NO
	41210	K57.2;K57.5	NO
	20002	1471;1483	YES
	20004	1524	YES
	40001	I48;I48.0;I48.1;I48.2;I48.3;I48.4;I48.9	YES
	40002	I48;I48.0;I48.1;I48.2;I48.3;I48.4;I48.9	YES
	41200	K57.1;K62.1;K62.2;K62.3;K62.4;X50.1;X50.2	YES
	41202	I48;I48.0;I48.1;I48.2;I48.3;I48.4;I48.9	YES
	41203	4273	YES
	41204	I48;I48.0;I48.1;I48.2;I48.3;I48.4;I48.9	YES
	41205	4273	YES
	41210	K57.1;K62.1;K62.2;K62.3;K62.4;X50.1;X50.2	YES
**Ventricular tachycardia**	40001	I47.0;I47.2;I49.0	NO
	40002	I47.0;I47.2;I49.0	NO
	41200	K57.6;K64.1	NO
	41202	I47.0;I47.2;I49.0	NO
	41203	4271;4274	NO
	41204	I47.0;I47.2;I49.0	NO
	41205	4271;4274	NO
	41210	K57.6;K64.1	NO

Note: 'Field' refers to the UK Biobank data field used to extract data; 'matching code' refers to the data coding, for instance ICD9 or ICD10 codes, used to define the disease from the respective field. 'Exclude' indicates whether the coding should be used to *exclude* the participant from the phenotype (instead of define the participant as a case based on the code).

Table III. Baseline characteristics for UK Biobank exome sequence cohort

Participants, N	454,162
Female, N (%)	246,303 (54.23)
European ancestry, N (%)	428,872 (94.4)
Age at enrollment, mean (SD)	57.04 (8.09)
Age at last follow-up, mean (SD)	68.00 (8.06)

Table IV. Case counts for UK Biobank exome sequence cohort

Disease endpoint	Cases, n (%)	Controls, n (%)
Dilated cardiomyopathy	924 (0.21)	438,722 (99.79)
Hypertrophic cardiomyopathy	538 (0.12)	453,624 (99.88)
Atrial fibrillation or flutter (AF)	29,149 (6.42)	425,013 (93.58)
Supraventricular tachycardia	4,836 (1.06)	449,326 (98.94)
Supraventricular tachycardia excluding AF	4,211 (1.07)	391,968 (99.53)
Ventricular tachycardia	2,297 (0.51)	451,865 (99.49)
Conduction system disease	12,331 (2.72)	441,831 (97.28)

Table V. TNNI3K mutagenesis primers. F: Forward, R: Reverse

TNNI3K variant			Sequence (5'-3')
c.1133G>A	p.Gly378Asp	F	TCTGCTCATCTTTTTCATCACTAGACCTGCTGGGA
		R	TCCCAGCAGGTCTAGTGATGAAAAAGATGAGCAGA
c.1349C>T	p.Ala450Val	F	GAAATGTGAAGGCAATCCAACTCTTAGGAGGAGAATATC
		R	GATATTCTCCTCCTAAGAGTTGGATTGCCTTCACATTTC
c.1469A>G	p.Lys490Arg	F	TTGGCTCGATAACGTCTTATAGCCACTATTTTATTTCTGCATCGT
		R	ACGATGCAGAAATAAAATAGTGGCTATAAGACGTTATCGAGCCAA
c.1528G>T	p.Val510Leu	F	CTGGCAGAGAATGGACAACTCTCGGCAAAACATAT
		R	ATATGTTTTGCCGAGAGTTGTCCATTCTCTGCCAG
c.1531T>C	p.Ser511Pro	F	GCTGGCAGAGAATGGGCACCTCTCGGCAAAA
		R	TTTTGCCGAGAGGTGCCCATTCTCTGCCAGC
c.1534A>T	p.Ile512Phe	F	GCCGAGAGGTGTCCTTTCTCTGCCAGCTC
		R	GAGCTGGCAGAGAAAGGACACCTCTCGGC
c.1535T>C	p.Ile512Thr	F	GCCGAGAGGTGTCCACTCTCTGCCAGCTC
		R	GAGCTGGCAGAGAGTGGACACCTCTCGGC
c.1722G>C	p.Met574Ile	F	GGTTGTGAAGGTACTCGATGCCTTTGGCAACATC
		R	GATGTTGCCAAAGGCATCGAGTACCTTCACAACC
c.1729C>T	p.Leu577Phe	F	CCAAAGGCATGGAGTACTTTCACAACCTGACACAG
		R	CTGTGTCAGGTTGTGAAAGTACTCCATGCCTTTGG
c.1772G>C	p.Ser591Thr	F	GAGAAGAATATTGTGAGTGTTCAAGTCACGATGTATAATTGGC
		R	GCCAATTATACATCGTGACTTGAACACTCACAATATTCTTCTC
	p.Arg556_Asn590del	F	GTTCTCCCTCCTTCATGAGCAGAAGAGTCACAATATTCTTCTCTATGAGGATGGGC
		R	GCCCATCCTCATAGAGAAGAATATTGTGACTCTTCTGCTCATGAAGGAGGGAGAAC
c.1774C>T	p.His592Tyr	F	CTCATAGAGAAGAATATTGTAACTGTTCAAGTCACGATGTATAATTGGC
		R	GCCAATTATACATCGTGACTTGAACAGTTACAATATTCTTCTCTATGAG
c.2012C>T	p.Ala671Val	F	TGCTGCCGCAGCCACTGGCTTGAGATG
		R	CATCTCAAGCCAGTGGCTGCGGCAGCA
c.2146G>C	p.Val716Leu	F	AAGACCCGAATTTTCTGAAGTTCTCATGAAGTTAGAAGAGTG
		R	CACTCTTCTAACTTCATGAGAACTTCAGAAAATTCGGGTCTT
c.2225C>T	p.Pro742Leu	F	CAGTGGGTCTCTCTCACTTTCTTCTTCTTCTGATTG
		R	CAATCAGAAGAAGAAGAAAGTGAGAGAGACCCACTG
c.2297G>C	p.Arg766Pro	F	AGCATATTCCAATTCGAAAGGACTTCTTAATGCTGCCAC
		R	GTGGCAGCATTAAGAAGTCCTTTCGAATTGGAATATGCT

Table VI. List of variants found in patients who underwent genetic screening of cardiomyopathy and/or arrhythmia panel

TNNI3K variant	Classification variants	Variant location	Auto-phosphorylation (relative to WT; p-value)	MAF	Allele count	Additional variants	Classification additional variants	Phenotype
c.271G>A; p.(Gly91Arg)	Class 3	Ankyrin 1		6,57E-06	1	*TNNI3K*-c.934G>A; p.(Ala312Thr)	Class 3	HCM, SVTs, SCD in the family
c.431C>T; p.(Ala144Val)	Class 3	Ankyrin 3		6,57E-06	1			HCM
c.797T>C; p.(Val266Ala)	Class 3	Ankyrin 6 - Ankyrin 7		0	1	*NEXN*-c.490-24_490del; p.(?)<br>*DES*-c.976C>T; p.(His326Tyr)	Class 3<br>Class 3	DCM
c.861G>T; p.(Lys287Asn)	Class 3	Ankyrin 7		0	1	*ACTN2*-c.690T>A; p.(Asp230Glu)	Class 3	HCM
c.934G>A; p.(Ala312Thr)	Class 3	Ankyrin 8		3,97E-05	1	*TNNI3K*-c.271G>A; p.(Gly91Arg)		HCM, SVTs, SCD in the family
c.1037C>A; p.(Ser346Tyr)	Class 3	Ankyrin 9		0	1			HCM
c.1133G>A; p.(Gly378Asp)	Class 3	Ankyrin 9 - Ankyrin 10	No change (1.1; 0.90)	6,57E-06	1	*HCN4*-c.3035G>C; p.(Gly1012Ala)<br>*ANK2*-c.6725C>T; p.(Thr2242Met)	Class 3<br>Class 3	VF, OHCA
c.1318A>C; p.(Lys440Gln)	Class 3	Ankyrin 10-Kinase domain		1,97E-05	1	*SCN5A*-c.1663G>A; p.(Glu555Lys)<br>*TTN*-c.11788G>A; p.(Glu3930Lys)<br>*TTN*-c.92696T>G; p.(Val30899Gly)	Class 3<br>Class 3<br>Class 3	DCM
c.1349C>T; p.(Ala450Val)	Class 3	Ankyrin 10-Kinase domain	No change (1.1; 0.97)	6,58E-06	3			CPVT
c.1349C>T; p.(Ala450Val)	Class 3<br>Class 3					*FLNC*-c.2068T>C; p.(Phe690Leu)<br>*JUP*-c.2128C>T; p.(Pro710Ser)	Class 3<br>Class 3	HCM
c.1442G>A; p.(Arg481Gln)	Class 3	Kinase domain		1,98E-05	1			OHCA, VF, susp CPVT
c.1535T>C; p.(Ile512Thr)	Class 3	Kinase domain	Increased (1.2; 0.01)	0,00E+00	0	*LAMA4*-c.2555C>T; p.(Thr852Met)	Class 3	See Supplemental Table XI for details
c.1535T>C; p.(Ile512Thr)								See Supplemental Table XI for details
c.1535T>C; p.(Ile512Thr)								See Supplemental Table XI for details
c.1535T>C; p.(Ile512Thr)						*LAMA4*-c.2555C>T; p.(Thr852Met)	Class 3	See Supplemental Table XI for details
c.1697T>C; p.(Ile566Thr)	Class 3	Kinase domain		6,58E-06	1			OHCA, VF
c.1722G>C; p.(Met574Ile)	Class 3	Kinase domain	No change (0.8; 0.29)	2,63E-05	3			NCCM
c.1774C>T; p.(His592Tyr)	**Class 4**	Kinase domain	Increased (1.2; 0.02)	3,69E-05	5			See Supplemental Table XI for details
c.1774C>T; p.(His592Tyr)								See Supplemental Table XI for details
c.1774C>T; p.(His592Tyr)								See Supplemental Table XI for details
c.1774C>T; p.(His592Tyr)								See Supplemental Table XI for details
c.1774C>T; p.(His592Tyr)								See Supplemental Table XI for details
c.1774C>T; p.(His592Tyr)						*RBM20*-c.3115C>T; p.(Pro1039Ser)	Class 3	See Supplemental Table XI for details
c.1774C>T; p.(His592Tyr)								See Supplemental Table XI for details
c.1774C>T; p.(His592Tyr)								See Supplemental Table XI for details
c.2012C>T; p.(Ala671Val)	Class 3	Kinase domain	Increased (1.7; <0.0001)	1,31E-05	2	*DSG2*-c.430G>A; p.(Glu144Lys)<br>*COL3A1*-c.3787A>G; p.(Arg1263Gly)	Class 3<br>Class 3	DCM, CCD
c.2018C>T; p.(Ala673Val)	Class 3	Kinase domain		2,63E-05	1	*TTN*-c.90955C>G; p.(Pro30319Ala)	Class 3	HCM, PVCs
c.2018C>A; p.(Ala673Glu)	Class 3	Kinase domain		4,60E-04	1	PKP2-c.2062T>C; p.(Ser688Pro)<br>*TTN*-c.47650T>C; p.(Ser15884Pro)	Class 3<br>Class 3	OHCA, ACM, SCD in the family
c.2146G>C; p.(Val716Leu)	Class 3	Kinase domain	No change (1.0; 1.0)	0	1	*TTN*-c.1297G>A; p.(Val433Ile)	Class 3	CM, atrial flutter, AT, LBBB
c.2164T>C; p.(Cys722Arg)	Class 3	Kinase domain		0	1	*LAMA4*-c.1634G>A; p.(Arg545His)	Class 3	DCM, arrhythmias
c.2203A>G; p.(Asn735Asp)	Class 3	Serine rich domain		0	1			HCM
c.2225C>T; p.(Pro742Leu)	Class 3	Serine rich domain	Decreased (0.6; 0.0014)	0	4	*PRKAG2*-c.1195C>T; p.(Leu399Phe)	Class 3	CJET
c.2225C>T; p.(Pro742Leu)	Class 3					FLNC-c.2345A>G; p.(Asn782Ser)	Class 3	DCM
c.2225C>T; p.(Pro742Leu)	Class 3					*TNNI3K*-c.1772G>C; p.(Arg556_Asn590del)/p.(Ser591Thr)	Class 3	DCM, VF
c.2254C>T; p.(Arg752Trp)	Class 3	C-terminus		1,97E-05	2			DCM
c.2255G>A; p.(Arg752Gln)	Class 3	C-terminus		1,97E-05	1			SSS
c.2269C>T; p.(Arg757Trp)	Class 3	C-terminus		1,32E-05	1			SCD, OHCA, VF
c.2297G>C; p.(Arg766Pro)	Class 3	C-terminus	No change (0.9; 1.0)	6,57E-05	3	*CDH2*-c.70G>C; p.(Glu24Gln)	Class 3	DCM
c.2297G>C; p.(Arg766Pro)	Class 3					*TTN*-c.80078del; p.(Pro26693Glnfs*10)<br>*TTN*-c.23203A>C; p.(Asn7735His)<br>*TTN*-c.46786T>C; p.(Tyr15596His)<br>*FLNC*-c.1568T>C; p.(Val523Ala)	**Class 4**<br>Class 3<br>Class 3<br>Class 3	DCM
c.2297G>C; p.(Arg766Pro)	Class 3							DCM, AF
c.2338G>A; p.(Ala780Thr)	Class 3	C-terminus		0	2	*TTN*-c.21836C>T; p.(Ala7279Val)	Class 3	DCM
c.2338G>A; p.(Ala780Thr)	Class 3							DCM
c.2365T>A; p.(Ser789Thr)	Class 3	C-terminus		0	1	*TTN*-c.40979G>A; p.(Arg13660His)<br>*TTN*-c.76280A>C; p.(Asn25427Thr)	Class 3<br>Class 3	DCM
c.2435A>G; p.(Tyr812Cys)	Class 3	C-terminus		0	1	*MYBPC3*-c.2479C>A; p.(Gln827Lys)<br>*SCN5A*-c.1844G>A; p.(Gly615Glu)<br>*MYPN*-c.140C>T; p.(Pro47Leu)	Class 3<br>Class 3<br>Class 3	HCM
c.2476C>G; p.(Arg826Gly)	Class 3	C-terminus		9,86E-05	1	*PRKAG2*-c.554A>T; p.(Glu185Val)<br>*TTN*-c.85463G>A; p.(Arg28488Gln)	Class 3<br>Class 3	NCCM, Ebstein syndrome
c.149+1G>A; p.(?)	Class 3	Coiled-coil		0	2	*KCNE1*-c.253G>A; p.(Asp85Asn)	Class 3	AF
c.149+1G>A; p.(?)	Class 3							DCM, NSVT
c.543+5_543+6delinsTA; p.(?)	Class 3	Ankyrin 4		0	1			VT/VF, OHCA
c.683-1G>A; p.(?)	Class 3	Ankyrin 5		6,57E-06		*TTN*-c.94024G>A; p.(Glu31342Lys)	Class 3	DCM
c.827+4A>C; p.(?) p.(Asn229Leufs*10)	Class 3<br>Class 3			2,69E-04	6	*TTN*-c.41559C>A; p.(Tyr13853X)<br>*LAMA4*-c.2576C>T; p.(Thr859Met)	**Class 5**<br>Class 3	DCM, WPW
c.827+4A>C; p.(?) p.(Asn229Leufs*10)	Class 3							DCM, SD
c.827+4A>C; p.(?) p.(Asn229Leufs*10)	Class 3					*DSP*-c.485G>A; p.(Arg162His)	Class 3	HCM
c.827+4A>C; p.(?) p.(Asn229Leufs*10)	Class 3							DCM, SCD in the family
c.933-1G>T; p.(?)	Class 3	Ankyrin 8		0	3	*MYH7*-c.4558G>A; p.(Gly1520Arg)	Class 3	SD
c.933-1G>T; p.(?)	Class 3					*TTN*-c.57199C>T; p.(Arg19067Cys)	Class 3	NSVT, ACM
c.933-1G>T; p.(?)	Class 3							HCM
c.1772G>C; p.(Arg556_Asn590del)/ p.(Ser591Thr) HOMOZYGOUS	Class 3	Kinase domain	Decreased (0.0; <0.0001)	1,09E-03	19	*FHL1*-c.944C>T; p.(Thr315Ile)<br>*TTN*-c.27243G>C; p.(Glu9081Asp)	Class 3<br>Class 3	Suspected CM
c.1772G>C; p.(Arg556_Asn590del)/ p.(Ser591Thr)	Class 3					*TTN*-c.96908A>C p.(Asp32303Ala)	Class 3	OHCA, VF, NSVT
c.1772G>C; p.(Arg556_Asn590del)/ p.(Ser591Thr)	Class 3					*KCNH2*-c.2127C>A; p.(Asn709Lys)<br>*MYH6*-c.3346C>A; p.(Arg1116Ser)	Class 3<br>Class 3	OHCA, VF
c.1772G>C; p.(Arg556_Asn590del)/ p.(Ser591Thr)	Class 3					*TTN*-c.1772G>C; p.(Glu11483Asp)	Class 3	Suspected ACM
c.1772G>C; p.(Arg556_Asn590del)/ p.(Ser591Thr)	Class 3					*TNNC1*-c.157A>G; p.(Thr53Ala)	Class 3	DCM, neuropathy
c.1772G>C; p.(Arg556_Asn590del)/ p.(Ser591Thr)	Class 3					*ALPK3*-c.4801G>C; p.(Gly1601Arg)	Class 3	ACM
c.1772G>C; p.(Arg556_Asn590del)/ p.(Ser591Thr)	Class 3					*PRDM16*-c.1882G>A; p.(Asp628Asn)	Class 3	DCM
c.1772G>C; p.(Arg556_Asn590del)/ p.(Ser591Thr)	Class 3							Broad complex SVTs
c.1772G>C; p.(Arg556_Asn590del)/ p.(Ser591Thr)	Class 3					*MYH7*-c.1291G>C; p.(Val431Leu)<br>*LAMA4*-c.1580T>C; p.(Met527Thr)<br>*TAZ*-c.779T>G; p.(Val260Gly)<br>*TTN*-c.47812G>A; p.(Asp15938Asn)<br>*TTN*-c.61160G>C; p.(Gly20387Ala)	Class 3<br>Class 3<br>Class 3<br>Class 3<br>Class 3	HCM, AF, AV block
c.1772G>C; p.(Arg556_Asn590del)/ p.(Ser591Thr)	Class 3							VF
c.1772G>C; p.(Arg556_Asn590del)/ p.(Ser591Thr)	Class 3					*MYH7*-c.5348A>C; p.(Lys1783Thr)	Class 3	HCM, AVNRT
c.1772G>C; p.(Arg556_Asn590del)/ p.(Ser591Thr)	Class 3					*TNNI3K*-c.2225C>T; p.(Pro742Leu)	Class 3	DCM, VF
c.1772G>C; p.(Arg556_Asn590del)/ p.(Ser591Thr)	Class 3					*MYBPC3*-c.1790G>A; p.(Cys566Trpfs*5)	**Class 5**	HCM, SCD in the family
c.1772G>C; p.(Arg556_Asn590del)/ p.(Ser591Thr)	Class 3					*TTN*-c.44630_44631del; p.(Val14877Glufs*3)<br>*TTN*-c.27442G>A; p.(Glu9148Lys)<br>*TTN*-c.36044C>G; p.(Thr12015Arg)	**Class 4**<br>Class 3<br>Class 3	DCM
c.1772G>C; p.(Arg556_Asn590del)/ p.(Ser591Thr)	Class 3							OHCA

ACM – arrhythmogenic cardiomyopathy; AVNRT – atrio-ventricular nodal reentry tachycardia; AVB – atrioventricular block; AF – atrial fibrillation; CJET – congenital junctional ectopic tachycardia; CPVT – catecholaminergic polymorphic ventricular tachycardia; DCM – dilated cardiomyopathy; JET – junction ectopic tachycardia; LBBB – left bundle branch block; LAD – left axis deviation; LAFB - left anterior fascicular block; LVEDD – left ventricular end-diastolic dimension; LVEF – left ventricular ejection fraction; MI – myocardial infarction; OHCA – out-of-hospital cardiac arrest; PVCs – premature cardiac contractions; RAD – right axis deviation; RBBB – right bundle branch block; RV – right ventricle; SCD – sudden cardiac death; SSS – sick sinus syndrome; SVT – supraventricular tachycardia; VT – ventricular tachycardia; WPW – Wolff-Parkinson-White

Table VII. Raw association results for rare TNNI3K variants and diseases in UK Biobank across all masks

Phenotype	Gene	Transcript	Frequency filter	Included variants	N variants	cMAC	Score	Score.SE	SPA *P*-value	SPA converged
Atrial fibrillation or flutter (AF)	ENSG00000116783	CANONICAL	MAF<0.1%	LOF+missense0.8	117	2126	13,597	11,018	0,217	NA
	ENSG00000116783	CANONICAL	MAF<0.1%	missense0.8	21	74	-0,558	1,788	0,755	NA
	ENSG00000116783	CANONICAL	MAF<0.1%	LOF+missense0.6	210	2755	23,445	12,598	0,063	NA
	ENSG00000116783	CANONICAL	MAF<0.1%	missense0.6	114	703	9,290	6,082	0,127	NA
	ENSG00000116783	CANONICAL	MAF<0.1%	LOF+missense0.4	380	5117	49,493	17,317	0,004	WAAR
	ENSG00000116783	CANONICAL	MAF<0.1%	missense0.4	284	3065	35,339	13,155	0,007	WAAR
	ENSG00000116783	CANONICAL	MAF<0.1%	LOF+missense0.2	564	6479	60,298	19,340	0,002	WAAR
	ENSG00000116783	CANONICAL	MAF<0.1%	missense0.2	468	4427	46,144	15,714	0,003	WAAR
	ENSG00000116783	CANONICAL	MAF<0.1%	LOF+missense0	636	7215	50,038	20,383	0,014	WAAR
	ENSG00000116783	CANONICAL	MAF<0.1%	missense0	540	5163	35,883	16,985	0,035	WAAR
	ENSG00000116783	CANONICAL	MAF<0.1%	LOF	96	2052	14,155	10,874	0,193	NA
	ENSG00000116783	CANONICAL	MAF<0.001%	LOF+missense0.8	69	147	-2,407	2,782	0,387	NA
	ENSG00000116783	CANONICAL	MAF<0.001%	missense0.8	13	31	0,483	1,163	0,678	NA
	ENSG00000116783	CANONICAL	MAF<0.001%	LOF+missense0.6	126	269	-1,258	3,922	0,748	NA
	ENSG00000116783	CANONICAL	MAF<0.001%	missense0.6	70	153	1,632	2,879	0,571	NA
	ENSG00000116783	CANONICAL	MAF<0.001%	LOF+missense0.4	232	462	2,786	5,185	0,591	NA
	ENSG00000116783	CANONICAL	MAF<0.001%	missense0.4	176	346	5,676	4,285	0,185	NA
	ENSG00000116783	CANONICAL	MAF<0.001%	LOF+missense0.2	353	702	3,390	6,240	0,587	NA
	ENSG00000116783	CANONICAL	MAF<0.001%	missense0.2	297	586	6,280	5,515	0,255	NA
	ENSG00000116783	CANONICAL	MAF<0.001%	LOF+missense0	395	795	4,378	6,596	0,507	NA
	ENSG00000116783	CANONICAL	MAF<0.001%	missense0	339	679	7,268	5,915	0,219	NA
	ENSG00000116783	CANONICAL	MAF<0.001%	LOF	56	116	-2,890	2,527	0,253	NA
	ENSG00000116783	CANONICAL	singletons	LOF+missense0.8	39	39	-2,110	1,370	0,124	NA
	ENSG00000116783	CANONICAL	singletons	LOF+missense0.6	66	66	-1,926	1,858	0,300	NA
	ENSG00000116783	CANONICAL	singletons	missense0.6	34	34	-0,064	1,345	0,962	NA
	ENSG00000116783	CANONICAL	singletons	LOF+missense0.4	127	127	-0,144	2,516	0,954	NA
	ENSG00000116783	CANONICAL	singletons	missense0.4	95	95	1,719	2,165	0,427	NA
	ENSG00000116783	CANONICAL	singletons	LOF+missense0.2	197	197	1,408	3,072	0,647	NA
	ENSG00000116783	CANONICAL	singletons	missense0.2	165	165	3,270	2,792	0,241	NA
	ENSG00000116783	CANONICAL	singletons	LOF+missense0	215	215	2,393	3,219	0,457	NA
	ENSG00000116783	CANONICAL	singletons	missense0	183	183	4,256	2,953	0,150	NA
	ENSG00000116783	CANONICAL	singletons	LOF	32	32	-1,863	1,283	0,146	NA
Conduction system disease	ENSG00000116783	CANONICAL	MAF<0.1%	LOF+missense0.8	117	2126	-4,046	7,430	0,586	NA
	ENSG00000116783	CANONICAL	MAF<0.1%	missense0.8	21	74	-1,652	1,255	0,188	NA
	ENSG00000116783	CANONICAL	MAF<0.1%	LOF+missense0.6	210	2755	1,598	8,510	0,851	NA
	ENSG00000116783	CANONICAL	MAF<0.1%	missense0.6	114	703	3,992	4,141	0,335	NA
	ENSG00000116783	CANONICAL	MAF<0.1%	LOF+missense0.4	380	5117	14,279	11,677	0,221	NA
	ENSG00000116783	CANONICAL	MAF<0.1%	missense0.4	284	3065	16,673	8,881	0,060	NA
	ENSG00000116783	CANONICAL	MAF<0.1%	LOF+missense0.2	564	6479	12,592	13,098	0,336	NA
	ENSG00000116783	CANONICAL	MAF<0.1%	missense0.2	468	4427	14,986	10,670	0,160	NA
	ENSG00000116783	CANONICAL	MAF<0.1%	LOF+missense0	636	7215	5,964	13,800	0,666	NA
	ENSG00000116783	CANONICAL	MAF<0.1%	missense0	540	5163	8,358	11,525	0,468	NA
	ENSG00000116783	CANONICAL	MAF<0.1%	LOF	96	2052	-2,394	7,324	0,744	NA
	ENSG00000116783	CANONICAL	MAF<0.001%	LOF+missense0.8	69	147	0,213	1,932	0,912	NA
	ENSG00000116783	CANONICAL	MAF<0.001%	missense0.8	13	31	-0,646	0,785	0,411	NA
	ENSG00000116783	CANONICAL	MAF<0.001%	LOF+missense0.6	126	269	-0,270	2,716	0,921	NA
	ENSG00000116783	CANONICAL	MAF<0.001%	missense0.6	70	153	-1,129	1,980	0,569	NA
	ENSG00000116783	CANONICAL	MAF<0.001%	LOF+missense0.4	232	462	-0,222	3,597	0,951	NA
	ENSG00000116783	CANONICAL	MAF<0.001%	missense0.4	176	346	-1,081	2,966	0,716	NA
	ENSG00000116783	CANONICAL	MAF<0.001%	LOF+missense0.2	353	702	1,789	4,322	0,679	NA
	ENSG00000116783	CANONICAL	MAF<0.001%	missense0.2	297	586	0,930	3,813	0,807	NA
	ENSG00000116783	CANONICAL	MAF<0.001%	LOF+missense0	395	795	2,445	4,578	0,593	NA
	ENSG00000116783	CANONICAL	MAF<0.001%	missense0	339	679	1,586	4,101	0,699	NA
	ENSG00000116783	CANONICAL	MAF<0.001%	LOF	56	116	0,859	1,765	0,626	NA
	ENSG00000116783	CANONICAL	singletons	LOF+missense0.8	39	39	1,046	0,953	0,273	NA
	ENSG00000116783	CANONICAL	singletons	LOF+missense0.6	66	66	0,224	1,296	0,863	NA
	ENSG00000116783	CANONICAL	singletons	missense0.6	34	34	-0,927	0,935	0,322	NA
	ENSG00000116783	CANONICAL	singletons	LOF+missense0.4	127	127	0,745	1,758	0,672	NA
	ENSG00000116783	CANONICAL	singletons	missense0.4	95	95	-0,406	1,511	0,788	NA
	ENSG00000116783	CANONICAL	singletons	LOF+missense0.2	197	197	0,115	2,155	0,957	NA
	ENSG00000116783	CANONICAL	singletons	missense0.2	165	165	-1,036	1,959	0,597	NA
	ENSG00000116783	CANONICAL	singletons	LOF+missense0	215	215	0,589	2,268	0,795	NA
	ENSG00000116783	CANONICAL	singletons	missense0	183	183	-0,562	2,083	0,787	NA
	ENSG00000116783	CANONICAL	singletons	LOF	32	32	1,151	0,898	0,200	NA
Hypertrophic cardiomyopathy	ENSG00000116783	CANONICAL	MAF<0.1%	LOF+missense0.8	117	2126	-2,527	1,588	0,112	NA
	ENSG00000116783	CANONICAL	MAF<0.1%	missense0.8	21	74	-0,081	0,283	0,776	NA
	ENSG00000116783	CANONICAL	MAF<0.1%	LOF+missense0.6	210	2755	-1,297	1,831	0,479	NA
	ENSG00000116783	CANONICAL	MAF<0.1%	missense0.6	114	703	1,150	0,921	0,212	NA
	ENSG00000116783	CANONICAL	MAF<0.1%	LOF+missense0.4	380	5117	-1,970	2,475	0,426	NA
	ENSG00000116783	CANONICAL	MAF<0.1%	missense0.4	284	3065	0,477	1,880	0,800	NA
	ENSG00000116783	CANONICAL	MAF<0.1%	LOF+missense0.2	564	6479	-2,778	2,821	0,325	NA
	ENSG00000116783	CANONICAL	MAF<0.1%	missense0.2	468	4427	-0,331	2,316	0,886	NA
	ENSG00000116783	CANONICAL	MAF<0.1%	LOF+missense0	636	7215	-3,657	2,973	0,219	NA
	ENSG00000116783	CANONICAL	MAF<0.1%	missense0	540	5163	-1,211	2,500	0,628	NA
	ENSG00000116783	CANONICAL	MAF<0.1%	LOF	96	2052	-2,447	1,563	0,117	NA
	ENSG00000116783	CANONICAL	MAF<0.001%	LOF+missense0.8	69	147	-0,197	0,447	0,660	NA
	ENSG00000116783	CANONICAL	MAF<0.001%	missense0.8	13	31	-0,028	0,168	0,866	NA
	ENSG00000116783	CANONICAL	MAF<0.001%	LOF+missense0.6	126	269	0,625	0,623	0,316	NA
	ENSG00000116783	CANONICAL	MAF<0.001%	missense0.6	70	153	0,794	0,453	0,080	NA
	ENSG00000116783	CANONICAL	MAF<0.001%	LOF+missense0.4	232	462	1,366	0,826	0,098	NA
	ENSG00000116783	CANONICAL	MAF<0.001%	missense0.4	176	346	1,535	0,689	0,038	WAAR
	ENSG00000116783	CANONICAL	MAF<0.001%	LOF+missense0.2	353	702	1,058	0,996	0,288	NA
	ENSG00000116783	CANONICAL	MAF<0.001%	missense0.2	297	586	1,227	0,886	0,166	NA
	ENSG00000116783	CANONICAL	MAF<0.001%	LOF+missense0	395	795	0,924	1,064	0,385	NA
	ENSG00000116783	CANONICAL	MAF<0.001%	missense0	339	679	1,093	0,962	0,256	NA
	ENSG00000116783	CANONICAL	MAF<0.001%	LOF	56	116	-0,168	0,414	0,684	NA
	ENSG00000116783	CANONICAL	singletons	LOF+missense0.8	39	39	-0,043	0,207	0,836	NA
	ENSG00000116783	CANONICAL	singletons	LOF+missense0.6	66	66	0,910	0,299	0,027	WAAR
	ENSG00000116783	CANONICAL	singletons	missense0.6	34	34	0,948	0,228	0,014	WAAR
	ENSG00000116783	CANONICAL	singletons	LOF+missense0.4	127	127	1,821	0,422	0,005	WAAR
	ENSG00000116783	CANONICAL	singletons	missense0.4	95	95	1,859	0,375	0,003	WAAR
	ENSG00000116783	CANONICAL	singletons	LOF+missense0.2	197	197	1,720	0,528	0,012	WAAR
	ENSG00000116783	CANONICAL	singletons	missense0.2	165	165	1,757	0,492	0,009	WAAR
	ENSG00000116783	CANONICAL	singletons	LOF+missense0	215	215	1,691	0,555	0,015	WAAR
	ENSG00000116783	CANONICAL	singletons	missense0	183	183	1,728	0,520	0,012	WAAR
	ENSG00000116783	CANONICAL	singletons	LOF	32	32	-0,037	0,193	0,846	NA

Dilated cardiomyopathy	ENSG00000116783	CANONICAL	MAF<0.1%	LOF+missense0.8	114	2058	-3,419	2,096	0,103	NA
	ENSG00000116783	CANONICAL	MAF<0.1%	missense0.8	20	71	-0,124	0,352	0,724	NA
	ENSG00000116783	CANONICAL	MAF<0.1%	LOF+missense0.6	207	2669	1,306	2,404	0,587	NA
	ENSG00000116783	CANONICAL	MAF<0.1%	missense0.6	113	682	4,600	1,180	0,002	WAAR
	ENSG00000116783	CANONICAL	MAF<0.1%	LOF+missense0.4	374	4956	7,474	3,279	0,022	WAAR
	ENSG00000116783	CANONICAL	MAF<0.1%	missense0.4	280	2969	10,769	2,495	0,000	WAAR
	ENSG00000116783	CANONICAL	MAF<0.1%	LOF+missense0.2	557	6264	8,631	3,690	0,019	WAAR
	ENSG00000116783	CANONICAL	MAF<0.1%	missense0.2	463	4277	11,925	3,014	0,000	WAAR
	ENSG00000116783	CANONICAL	MAF<0.1%	LOF+missense0	627	6977	9,112	3,891	0,019	WAAR
	ENSG00000116783	CANONICAL	MAF<0.1%	missense0	533	4990	12,407	3,258	0,000	WAAR
	ENSG00000116783	CANONICAL	MAF<0.1%	LOF	94	1987	-3,294	2,066	0,111	NA
	ENSG00000116783	CANONICAL	MAF<0.001%	LOF+missense0.8	67	135	-0,273	0,527	0,605	NA
	ENSG00000116783	CANONICAL	MAF<0.001%	missense0.8	12	22	-0,030	0,173	0,863	NA
	ENSG00000116783	CANONICAL	MAF<0.001%	LOF+missense0.6	124	254	2,455	0,759	0,010	WAAR
	ENSG00000116783	CANONICAL	MAF<0.001%	missense0.6	69	141	2,698	0,548	0,001	WAAR
	ENSG00000116783	CANONICAL	MAF<0.001%	LOF+missense0.4	226	427	3,097	0,990	0,010	WAAR
	ENSG00000116783	CANONICAL	MAF<0.001%	missense0.4	171	314	3,340	0,817	0,002	WAAR
	ENSG00000116783	CANONICAL	MAF<0.001%	LOF+missense0.2	345	643	3,689	1,180	0,008	WAAR
	ENSG00000116783	CANONICAL	MAF<0.001%	missense0.2	290	530	3,932	1,039	0,003	WAAR
	ENSG00000116783	CANONICAL	MAF<0.001%	LOF+missense0	385	732	3,494	1,262	0,013	WAAR
	ENSG00000116783	CANONICAL	MAF<0.001%	missense0	330	619	3,737	1,132	0,005	WAAR
	ENSG00000116783	CANONICAL	MAF<0.001%	LOF	55	113	-0,243	0,498	0,626	NA
	ENSG00000116783	CANONICAL	singletons	LOF+missense0.8	38	38	-0,068	0,260	0,794	NA
	ENSG00000116783	CANONICAL	singletons	LOF+missense0.6	65	65	-0,138	0,370	0,709	NA
	ENSG00000116783	CANONICAL	singletons	missense0.6	34	34	-0,079	0,280	0,778	NA
	ENSG00000116783	CANONICAL	singletons	LOF+missense0.4	125	125	-0,262	0,510	0,608	NA
	ENSG00000116783	CANONICAL	singletons	missense0.4	94	94	-0,203	0,449	0,652	NA
	ENSG00000116783	CANONICAL	singletons	LOF+missense0.2	195	195	-0,403	0,633	0,524	NA
	ENSG00000116783	CANONICAL	singletons	missense0.2	164	164	-0,343	0,584	0,557	NA
	ENSG00000116783	CANONICAL	singletons	LOF+missense0	211	211	-0,438	0,660	0,507	NA
	ENSG00000116783	CANONICAL	singletons	missense0	180	180	-0,379	0,613	0,537	NA
	ENSG00000116783	CANONICAL	singletons	LOF	31	31	-0,059	0,243	0,807	NA
Supraventricular tachycardia	ENSG00000116783	CANONICAL	MAF<0.1%	LOF+missense0.8	117	2126	7,458	4,717	0,114	NA
	ENSG00000116783	CANONICAL	MAF<0.1%	missense0.8	21	74	0,319	0,820	0,697	NA
	ENSG00000116783	CANONICAL	MAF<0.1%	LOF+missense0.6	210	2755	9,958	5,417	0,066	NA
	ENSG00000116783	CANONICAL	MAF<0.1%	missense0.6	114	703	2,819	2,663	0,290	NA
	ENSG00000116783	CANONICAL	MAF<0.1%	LOF+missense0.4	380	5117	17,739	7,433	0,017	WAAR
	ENSG00000116783	CANONICAL	MAF<0.1%	missense0.4	284	3065	10,601	5,669	0,061	NA
	ENSG00000116783	CANONICAL	MAF<0.1%	LOF+missense0.2	564	6479	18,545	8,326	0,026	WAAR
	ENSG00000116783	CANONICAL	MAF<0.1%	missense0.2	468	4427	11,407	6,794	0,093	NA
	ENSG00000116783	CANONICAL	MAF<0.1%	LOF+missense0	636	7215	16,799	8,776	0,056	NA
	ENSG00000116783	CANONICAL	MAF<0.1%	missense0	540	5163	9,660	7,341	0,188	NA
	ENSG00000116783	CANONICAL	MAF<0.1%	LOF	96	2052	7,139	4,646	0,124	NA
	ENSG00000116783	CANONICAL	MAF<0.001%	LOF+missense0.8	69	147	-0,506	1,240	0,683	NA
	ENSG00000116783	CANONICAL	MAF<0.001%	missense0.8	13	31	0,716	0,530	0,177	NA
	ENSG00000116783	CANONICAL	MAF<0.001%	LOF+missense0.6	126	269	-0,783	1,697	0,645	NA
	ENSG00000116783	CANONICAL	MAF<0.001%	missense0.6	70	153	0,440	1,241	0,723	NA
	ENSG00000116783	CANONICAL	MAF<0.001%	LOF+missense0.4	232	462	-0,747	2,263	0,741	NA
	ENSG00000116783	CANONICAL	MAF<0.001%	missense0.4	176	346	0,475	1,880	0,801	NA
	ENSG00000116783	CANONICAL	MAF<0.001%	LOF+missense0.2	353	702	2,819	2,748	0,305	NA
	ENSG00000116783	CANONICAL	MAF<0.001%	missense0.2	297	586	4,041	2,444	0,098	NA
	ENSG00000116783	CANONICAL	MAF<0.001%	LOF+missense0	395	795	4,927	2,907	0,090	NA
	ENSG00000116783	CANONICAL	MAF<0.001%	missense0	339	679	6,149	2,620	0,019	WAAR
	ENSG00000116783	CANONICAL	MAF<0.001%	LOF	56	116	-1,223	1,121	0,275	NA
	ENSG00000116783	CANONICAL	singletons	LOF+missense0.8	39	39	-0,378	0,611	0,536	NA
	ENSG00000116783	CANONICAL	singletons	LOF+missense0.6	66	66	-0,653	0,803	0,416	NA
	ENSG00000116783	CANONICAL	singletons	missense0.6	34	34	-0,332	0,573	0,562	NA
	ENSG00000116783	CANONICAL	singletons	LOF+missense0.4	127	127	-1,222	1,099	0,266	NA
	ENSG00000116783	CANONICAL	singletons	missense0.4	95	95	-0,901	0,943	0,339	NA
	ENSG00000116783	CANONICAL	singletons	LOF+missense0.2	197	197	0,101	1,369	0,941	NA
	ENSG00000116783	CANONICAL	singletons	missense0.2	165	165	0,422	1,248	0,735	NA
	ENSG00000116783	CANONICAL	singletons	LOF+missense0	215	215	0,927	1,431	0,517	NA
	ENSG00000116783	CANONICAL	singletons	missense0	183	183	1,248	1,315	0,343	NA
	ENSG00000116783	CANONICAL	singletons	LOF	32	32	-0,321	0,563	0,569	NA
Supraventricular tachycardia excluding AF	ENSG00000116783	CANONICAL	MAF<0.1%	LOF+missense0.8	112	1806	2,574	2,898	0,375	NA
	ENSG00000116783	CANONICAL	MAF<0.1%	missense0.8	19	67	-0,303	0,549	0,581	NA
	ENSG00000116783	CANONICAL	MAF<0.1%	LOF+missense0.6	201	2328	4,140	3,328	0,214	NA
	ENSG00000116783	CANONICAL	MAF<0.1%	missense0.6	108	589	1,263	1,651	0,444	NA
	ENSG00000116783	CANONICAL	MAF<0.1%	LOF+missense0.4	364	4354	2,579	4,579	0,573	NA
	ENSG00000116783	CANONICAL	MAF<0.1%	missense0.4	271	2615	-0,298	3,507	0,932	NA
	ENSG00000116783	CANONICAL	MAF<0.1%	LOF+missense0.2	540	5547	2,973	5,154	0,564	NA
	ENSG00000116783	CANONICAL	MAF<0.1%	missense0.2	447	3808	0,096	4,227	0,982	NA
	ENSG00000116783	CANONICAL	MAF<0.1%	LOF+missense0	608	6184	2,001	5,436	0,713	NA
	ENSG00000116783	CANONICAL	MAF<0.1%	missense0	515	4445	-0,875	4,568	0,848	NA
	ENSG00000116783	CANONICAL	MAF<0.1%	LOF	93	1739	2,876	2,846	0,312	NA
	ENSG00000116783	CANONICAL	MAF<0.001%	LOF+missense0.8	64	119	-0,571	0,769	0,458	NA
	ENSG00000116783	CANONICAL	MAF<0.001%	LOF+missense0.6	118	222	-1,036	1,039	0,319	NA
	ENSG00000116783	CANONICAL	MAF<0.001%	missense0.6	64	121	-0,550	0,740	0,457	NA
	ENSG00000116783	CANONICAL	MAF<0.001%	LOF+missense0.4	216	382	0,226	1,395	0,871	NA
	ENSG00000116783	CANONICAL	MAF<0.001%	missense0.4	162	281	0,713	1,146	0,534	NA
	ENSG00000116783	CANONICAL	MAF<0.001%	LOF+missense0.2	327	571	0,319	1,690	0,850	NA
	ENSG00000116783	CANONICAL	MAF<0.001%	missense0.2	273	470	0,806	1,491	0,589	NA
	ENSG00000116783	CANONICAL	MAF<0.001%	LOF+missense0	366	649	0,981	1,790	0,583	NA
	ENSG00000116783	CANONICAL	MAF<0.001%	missense0	312	548	1,468	1,603	0,360	NA
	ENSG00000116783	CANONICAL	MAF<0.001%	LOF	54	101	-0,486	0,712	0,494	NA
	ENSG00000116783	CANONICAL	singletons	LOF+missense0.8	38	38	-0,177	0,420	0,673	NA
	ENSG00000116783	CANONICAL	singletons	LOF+missense0.6	69	69	-0,311	0,556	0,576	NA
	ENSG00000116783	CANONICAL	singletons	missense0.6	37	37	-0,161	0,400	0,688	NA
	ENSG00000116783	CANONICAL	singletons	LOF+missense0.4	129	129	-0,566	0,750	0,451	NA
	ENSG00000116783	CANONICAL	singletons	missense0.4	97	97	-0,416	0,643	0,518	NA
	ENSG00000116783	CANONICAL	singletons	LOF+missense0.2	197	197	0,105	0,949	0,912	NA
	ENSG00000116783	CANONICAL	singletons	missense0.2	165	165	0,255	0,867	0,768	NA
	ENSG00000116783	CANONICAL	singletons	LOF+missense0	216	216	0,021	0,992	0,983	NA
	ENSG00000116783	CANONICAL	singletons	missense0	184	184	0,171	0,913	0,851	NA
	ENSG00000116783	CANONICAL	singletons	LOF	32	32	-0,150	0,386	0,698	NA

Ventricular tachycardia	ENSG00000116783	CANONICAL	MAF<0.1%	LOF+missense0.8	117	2126	0,209	3,266	0,949	NA
	ENSG00000116783	CANONICAL	MAF<0.1%	missense0.8	21	74	0,688	0,556	0,215	NA
	ENSG00000116783	CANONICAL	MAF<0.1%	LOF+missense0.6	210	2755	5,080	3,749	0,175	NA
	ENSG00000116783	CANONICAL	MAF<0.1%	missense0.6	114	703	5,559	1,848	0,005	WAAR
	ENSG00000116783	CANONICAL	MAF<0.1%	LOF+missense0.4	380	5117	7,350	5,115	0,151	NA
	ENSG00000116783	CANONICAL	MAF<0.1%	missense0.4	284	3065	7,829	3,886	0,043	WAAR
	ENSG00000116783	CANONICAL	MAF<0.1%	LOF+missense0.2	564	6479	4,424	5,752	0,442	NA
	ENSG00000116783	CANONICAL	MAF<0.1%	missense0.2	468	4427	4,903	4,688	0,296	NA
	ENSG00000116783	CANONICAL	MAF<0.1%	LOF+missense0	636	7215	2,714	6,064	0,654	NA
	ENSG00000116783	CANONICAL	MAF<0.1%	missense0	540	5163	3,194	5,069	0,529	NA
	ENSG00000116783	CANONICAL	MAF<0.1%	LOF	96	2052	-0,479	3,219	0,882	NA
	ENSG00000116783	CANONICAL	MAF<0.001%	LOF+missense0.8	69	147	0,361	0,805	0,654	NA
	ENSG00000116783	CANONICAL	MAF<0.001%	missense0.8	13	31	0,885	0,337	0,036	WAAR
	ENSG00000116783	CANONICAL	MAF<0.001%	LOF+missense0.6	126	269	0,723	1,159	0,533	NA
	ENSG00000116783	CANONICAL	MAF<0.001%	missense0.6	70	153	1,248	0,863	0,148	NA
	ENSG00000116783	CANONICAL	MAF<0.001%	LOF+missense0.4	232	462	0,773	1,556	0,619	NA
	ENSG00000116783	CANONICAL	MAF<0.001%	missense0.4	176	346	1,298	1,304	0,320	NA
	ENSG00000116783	CANONICAL	MAF<0.001%	LOF+missense0.2	353	702	1,632	1,888	0,388	NA
	ENSG00000116783	CANONICAL	MAF<0.001%	missense0.2	297	586	2,157	1,687	0,201	NA
	ENSG00000116783	CANONICAL	MAF<0.001%	LOF+missense0	395	795	2,178	2,004	0,277	NA
	ENSG00000116783	CANONICAL	MAF<0.001%	missense0	339	679	2,703	1,816	0,137	NA
	ENSG00000116783	CANONICAL	MAF<0.001%	LOF	56	116	-0,525	0,731	0,473	NA
	ENSG00000116783	CANONICAL	singletons	LOF+missense0.8	39	39	-0,161	0,400	0,687	NA
	ENSG00000116783	CANONICAL	singletons	LOF+missense0.6	66	66	-0,310	0,555	0,576	NA
	ENSG00000116783	CANONICAL	singletons	missense0.6	34	34	-0,167	0,407	0,681	NA
	ENSG00000116783	CANONICAL	singletons	LOF+missense0.4	127	127	-0,612	0,779	0,432	NA
	ENSG00000116783	CANONICAL	singletons	missense0.4	95	95	-0,469	0,681	0,491	NA
	ENSG00000116783	CANONICAL	singletons	LOF+missense0.2	197	197	0,085	0,952	0,929	NA
	ENSG00000116783	CANONICAL	singletons	missense0.2	165	165	0,229	0,874	0,794	NA
	ENSG00000116783	CANONICAL	singletons	LOF+missense0	215	215	-0,013	1,001	0,990	NA
	ENSG00000116783	CANONICAL	singletons	missense0	183	183	0,130	0,928	0,888	NA
	ENSG00000116783	CANONICAL	singletons	LOF	32	32	-0,143	0,377	0,704	NA

Note: abbreviations: cMAC, cumulative minor allele count; SE, standard error; SPA, saddle-point approximation; MAF, minor allele count; LOF, loss-of-function; NA, not applicable

Table VIII. Raw association results for rare TNNI3K variants and diseases in UK Biobank restricting to European ancestry samples

Phenotype	Gene	Transcript	Frequency filter	Included variants	N variants	cMAC	Score	Score.SE	SPA *P*-value	SPA converged
Atrial fibrillation or flutter (AF)	ENSG00000116783	CANONICAL	MAF<0.1%	LOF+missense0.8	93	2022	13,824	10,880	0,204	NA
	ENSG00000116783	CANONICAL	MAF<0.1%	missense0.8	18	61	-0,079	1,661	0,962	NA
	ENSG00000116783	CANONICAL	MAF<0.1%	LOF+missense0.6	172	2589	23,821	12,399	0,055	NA
	ENSG00000116783	CANONICAL	MAF<0.1%	missense0.6	97	628	9,919	5,877	0,091	NA
	ENSG00000116783	CANONICAL	MAF<0.1%	LOF+missense0.4	311	4850	48,590	17,095	0,004	WAAR
	ENSG00000116783	CANONICAL	MAF<0.1%	missense0.4	236	2889	34,687	12,961	0,007	WAAR
	ENSG00000116783	CANONICAL	MAF<0.1%	LOF+missense0.2	466	6021	58,992	18,971	0,002	WAAR
	ENSG00000116783	CANONICAL	MAF<0.1%	missense0.2	391	4060	45,090	15,349	0,003	WAAR
	ENSG00000116783	CANONICAL	MAF<0.1%	LOF+missense0	525	6703	49,694	19,986	0,013	WAAR
	ENSG00000116783	CANONICAL	MAF<0.1%	missense0	450	4742	35,792	16,591	0,031	WAAR
	ENSG00000116783	CANONICAL	MAF<0.1%	LOF	75	1961	13,903	10,754	0,196	NA
	ENSG00000116783	CANONICAL	MAF<0.001%	LOF+missense0.8	57	114	-2,955	2,536	0,244	NA
	ENSG00000116783	CANONICAL	MAF<0.001%	missense0.8	11	21	0,208	0,857	0,808	NA
	ENSG00000116783	CANONICAL	MAF<0.001%	LOF+missense0.6	107	221	-1,152	3,675	0,754	NA
	ENSG00000116783	CANONICAL	MAF<0.001%	missense0.6	61	128	2,011	2,662	0,450	NA
	ENSG00000116783	CANONICAL	MAF<0.001%	LOF+missense0.4	195	375	1,316	4,862	0,787	NA
	ENSG00000116783	CANONICAL	MAF<0.001%	missense0.4	149	282	4,479	3,980	0,260	NA
	ENSG00000116783	CANONICAL	MAF<0.001%	LOF+missense0.2	296	569	3,062	5,809	0,598	NA
	ENSG00000116783	CANONICAL	MAF<0.001%	missense0.2	250	476	6,225	5,094	0,222	NA
	ENSG00000116783	CANONICAL	MAF<0.001%	LOF+missense0	326	642	3,853	6,127	0,529	NA
	ENSG00000116783	CANONICAL	MAF<0.001%	missense0	280	549	7,016	5,454	0,198	NA
	ENSG00000116783	CANONICAL	MAF<0.001%	LOF	46	93	-3,163	2,387	0,185	NA
	ENSG00000116783	CANONICAL	singletons	LOF+missense0.8	32	32	-1,872	1,286	0,145	NA
	ENSG00000116783	CANONICAL	singletons	LOF+missense0.6	55	55	-1,548	1,764	0,380	NA
	ENSG00000116783	CANONICAL	singletons	missense0.6	29	29	0,094	1,294	0,942	NA
	ENSG00000116783	CANONICAL	singletons	LOF+missense0.4	103	103	0,599	2,378	0,801	NA
	ENSG00000116783	CANONICAL	singletons	missense0.4	77	77	2,241	2,053	0,275	NA
	ENSG00000116783	CANONICAL	singletons	LOF+missense0.2	159	159	2,638	2,883	0,360	NA
	ENSG00000116783	CANONICAL	singletons	missense0.2	133	133	4,279	2,622	0,103	NA
	ENSG00000116783	CANONICAL	singletons	LOF+missense0	170	170	3,929	2,992	0,189	NA
	ENSG00000116783	CANONICAL	singletons	missense0	144	144	5,571	2,742	0,040	WAAR
	ENSG00000116783	CANONICAL	singletons	LOF	26	26	-1,641	1,199	0,171	NA
Conduction system disease	ENSG00000116783	CANONICAL	MAF<0.1%	LOF+missense0.8	93	2022	-2,655	7,275	0,715	NA
	ENSG00000116783	CANONICAL	MAF<0.1%	missense0.8	18	61	-1,306	1,118	0,243	NA
	ENSG00000116783	CANONICAL	MAF<0.1%	LOF+missense0.6	172	2589	2,525	8,285	0,761	NA
	ENSG00000116783	CANONICAL	MAF<0.1%	missense0.6	97	628	3,875	3,918	0,323	NA
	ENSG00000116783	CANONICAL	MAF<0.1%	LOF+missense0.4	311	4850	16,231	11,428	0,156	NA
	ENSG00000116783	CANONICAL	MAF<0.1%	missense0.4	236	2889	17,580	8,666	0,042	WAAR
	ENSG00000116783	CANONICAL	MAF<0.1%	LOF+missense0.2	466	6021	14,433	12,683	0,255	NA
	ENSG00000116783	CANONICAL	MAF<0.1%	missense0.2	391	4060	15,783	10,264	0,124	NA
	ENSG00000116783	CANONICAL	MAF<0.1%	LOF+missense0	525	6703	8,207	13,356	0,539	NA
	ENSG00000116783	CANONICAL	MAF<0.1%	missense0	450	4742	9,557	11,088	0,389	NA
	ENSG00000116783	CANONICAL	MAF<0.1%	LOF	75	1961	-1,350	7,190	0,851	NA
	ENSG00000116783	CANONICAL	MAF<0.001%	LOF+missense0.8	57	114	1,074	1,706	0,529	NA
	ENSG00000116783	CANONICAL	MAF<0.001%	missense0.8	11	21	-0,323	0,561	0,565	NA
	ENSG00000116783	CANONICAL	MAF<0.001%	LOF+missense0.6	107	221	0,057	2,474	0,982	NA
	ENSG00000116783	CANONICAL	MAF<0.001%	missense0.6	61	128	-1,340	1,784	0,453	NA
	ENSG00000116783	CANONICAL	MAF<0.001%	LOF+missense0.4	195	375	1,020	3,280	0,756	NA
	ENSG00000116783	CANONICAL	MAF<0.001%	missense0.4	149	282	-0,377	2,678	0,888	NA
	ENSG00000116783	CANONICAL	MAF<0.001%	LOF+missense0.2	296	569	1,269	3,916	0,746	NA
	ENSG00000116783	CANONICAL	MAF<0.001%	missense0.2	250	476	-0,128	3,427	0,970	NA
	ENSG00000116783	CANONICAL	MAF<0.001%	LOF+missense0	326	642	1,509	4,125	0,714	NA
	ENSG00000116783	CANONICAL	MAF<0.001%	missense0	280	549	0,112	3,665	0,976	NA
	ENSG00000116783	CANONICAL	MAF<0.001%	LOF	46	93	1,397	1,611	0,386	NA
	ENSG00000116783	CANONICAL	singletons	LOF+missense0.8	32	32	1,215	0,864	0,159	NA
	ENSG00000116783	CANONICAL	singletons	LOF+missense0.6	55	55	0,515	1,187	0,665	NA
	ENSG00000116783	CANONICAL	singletons	missense0.6	29	29	-0,796	0,870	0,360	NA
	ENSG00000116783	CANONICAL	singletons	LOF+missense0.4	103	103	1,305	1,602	0,415	NA
	ENSG00000116783	CANONICAL	singletons	missense0.4	77	77	-0,006	1,383	0,996	NA
	ENSG00000116783	CANONICAL	singletons	LOF+missense0.2	159	159	0,052	1,939	0,979	NA
	ENSG00000116783	CANONICAL	singletons	missense0.2	133	133	-1,259	1,763	0,475	NA
	ENSG00000116783	CANONICAL	singletons	LOF+missense0	170	170	-0,248	2,012	0,902	NA
	ENSG00000116783	CANONICAL	singletons	missense0	144	144	-1,559	1,843	0,398	NA
	ENSG00000116783	CANONICAL	singletons	LOF	26	26	1,311	0,808	0,105	NA
Hypertrophic cardiomyopathy	ENSG00000116783	CANONICAL	MAF<0.1%	LOF+missense0.8	93	2022	-2,251	1,497	0,133	NA
	ENSG00000116783	CANONICAL	MAF<0.1%	missense0.8	18	61	-0,063	0,250	0,802	NA
	ENSG00000116783	CANONICAL	MAF<0.1%	LOF+missense0.6	172	2589	-1,866	1,708	0,275	NA
	ENSG00000116783	CANONICAL	MAF<0.1%	missense0.6	97	628	0,322	0,822	0,695	NA
	ENSG00000116783	CANONICAL	MAF<0.1%	LOF+missense0.4	311	4850	-2,328	2,341	0,320	NA
	ENSG00000116783	CANONICAL	MAF<0.1%	missense0.4	236	2889	-0,140	1,776	0,937	NA
	ENSG00000116783	CANONICAL	MAF<0.1%	LOF+missense0.2	466	6021	-2,632	2,605	0,312	NA
	ENSG00000116783	CANONICAL	MAF<0.1%	missense0.2	391	4060	-0,444	2,111	0,834	NA
	ENSG00000116783	CANONICAL	MAF<0.1%	LOF+missense0	525	6703	-3,381	2,744	0,218	NA
	ENSG00000116783	CANONICAL	MAF<0.1%	missense0	450	4742	-1,193	2,282	0,601	NA
	ENSG00000116783	CANONICAL	MAF<0.1%	LOF	75	1961	-2,188	1,476	0,138	NA
	ENSG00000116783	CANONICAL	MAF<0.001%	LOF+missense0.8	57	114	-0,116	0,345	0,737	NA
	ENSG00000116783	CANONICAL	MAF<0.001%	missense0.8	11	21	-0,016	0,126	0,899	NA
	ENSG00000116783	CANONICAL	MAF<0.001%	LOF+missense0.6	107	221	-0,245	0,510	0,631	NA
	ENSG00000116783	CANONICAL	MAF<0.001%	missense0.6	61	128	-0,145	0,380	0,703	NA
	ENSG00000116783	CANONICAL	MAF<0.001%	LOF+missense0.4	195	375	0,569	0,695	0,413	NA
	ENSG00000116783	CANONICAL	MAF<0.001%	missense0.4	149	282	0,669	0,588	0,255	NA
	ENSG00000116783	CANONICAL	MAF<0.001%	LOF+missense0.2	296	569	0,354	0,837	0,672	NA
	ENSG00000116783	CANONICAL	MAF<0.001%	missense0.2	250	476	0,454	0,751	0,545	NA
	ENSG00000116783	CANONICAL	MAF<0.001%	LOF+missense0	326	642	0,268	0,887	0,763	NA
	ENSG00000116783	CANONICAL	MAF<0.001%	missense0	280	549	0,368	0,806	0,648	NA
	ENSG00000116783	CANONICAL	MAF<0.001%	LOF	46	93	-0,100	0,322	0,756	NA
	ENSG00000116783	CANONICAL	singletons	LOF+missense0.8	32	32	-0,031	0,177	0,860	NA
	ENSG00000116783	CANONICAL	singletons	LOF+missense0.6	55	55	-0,058	0,240	0,810	NA
	ENSG00000116783	CANONICAL	singletons	missense0.6	29	29	-0,031	0,176	0,860	NA
	ENSG00000116783	CANONICAL	singletons	LOF+missense0.4	103	103	0,876	0,351	0,040	WAAR
	ENSG00000116783	CANONICAL	singletons	missense0.4	77	77	0,903	0,311	0,030	WAAR
	ENSG00000116783	CANONICAL	singletons	LOF+missense0.2	159	159	0,821	0,422	0,052	NA
	ENSG00000116783	CANONICAL	singletons	missense0.2	133	133	0,848	0,390	0,052	WAAR
	ENSG00000116783	CANONICAL	singletons	LOF+missense0	170	170	0,804	0,442	0,069	NA
	ENSG00000116783	CANONICAL	singletons	missense0	144	144	0,831	0,410	0,059	WAAR
	ENSG00000116783	CANONICAL	singletons	LOF	26	26	-0,027	0,164	0,870	NA

Dilated cardiomyopathy	ENSG00000116783	CANONICAL	MAF<0.1%	LOF+missense0.8	91	1957	-3,176	2,036	0,119	NA
	ENSG00000116783	CANONICAL	MAF<0.1%	missense0.8	17	58	-0,098	0,312	0,754	NA
	ENSG00000116783	CANONICAL	MAF<0.1%	LOF+missense0.6	170	2509	0,700	2,318	0,763	NA
	ENSG00000116783	CANONICAL	MAF<0.1%	missense0.6	96	610	3,778	1,102	0,004	WAAR
	ENSG00000116783	CANONICAL	MAF<0.1%	LOF+missense0.4	306	4699	6,072	3,183	0,056	NA
	ENSG00000116783	CANONICAL	MAF<0.1%	missense0.4	232	2800	9,150	2,416	0,001	WAAR
	ENSG00000116783	CANONICAL	MAF<0.1%	LOF+missense0.2	460	5822	7,736	3,531	0,028	WAAR
	ENSG00000116783	CANONICAL	MAF<0.1%	missense0.2	386	3923	10,814	2,858	0,001	WAAR
	ENSG00000116783	CANONICAL	MAF<0.1%	LOF+missense0	519	6483	8,348	3,722	0,024	WAAR
	ENSG00000116783	CANONICAL	MAF<0.1%	missense0	445	4584	11,426	3,092	0,001	WAAR
	ENSG00000116783	CANONICAL	MAF<0.1%	LOF	74	1899	-3,078	2,012	0,126	NA
	ENSG00000116783	CANONICAL	MAF<0.001%	LOF+missense0.8	57	112	-0,206	0,460	0,653	NA
	ENSG00000116783	CANONICAL	MAF<0.001%	missense0.8	11	21	-0,028	0,166	0,868	NA
	ENSG00000116783	CANONICAL	MAF<0.001%	LOF+missense0.6	107	217	2,568	0,680	0,006	WAAR
	ENSG00000116783	CANONICAL	MAF<0.001%	missense0.6	61	126	2,747	0,502	0,001	WAAR
	ENSG00000116783	CANONICAL	MAF<0.001%	LOF+missense0.4	192	362	2,279	0,887	0,023	WAAR
	ENSG00000116783	CANONICAL	MAF<0.001%	missense0.4	146	271	2,457	0,738	0,008	WAAR
	ENSG00000116783	CANONICAL	MAF<0.001%	LOF+missense0.2	293	552	2,944	1,061	0,015	WAAR
	ENSG00000116783	CANONICAL	MAF<0.001%	missense0.2	247	461	3,123	0,940	0,006	WAAR
	ENSG00000116783	CANONICAL	MAF<0.001%	LOF+missense0	323	623	2,800	1,127	0,021	WAAR
	ENSG00000116783	CANONICAL	MAF<0.001%	missense0	277	532	2,979	1,014	0,010	WAAR
	ENSG00000116783	CANONICAL	MAF<0.001%	LOF	46	91	-0,179	0,429	0,677	NA
	ENSG00000116783	CANONICAL	singletons	LOF+missense0.8	32	32	-0,057	0,237	0,811	NA
	ENSG00000116783	CANONICAL	singletons	LOF+missense0.6	55	55	-0,109	0,329	0,741	NA
	ENSG00000116783	CANONICAL	singletons	missense0.6	29	29	-0,059	0,243	0,807	NA
	ENSG00000116783	CANONICAL	singletons	LOF+missense0.4	101	101	-0,205	0,452	0,650	NA
	ENSG00000116783	CANONICAL	singletons	missense0.4	75	75	-0,156	0,394	0,692	NA
	ENSG00000116783	CANONICAL	singletons	LOF+missense0.2	157	157	-0,296	0,543	0,585	NA
	ENSG00000116783	CANONICAL	singletons	missense0.2	131	131	-0,247	0,496	0,618	NA
	ENSG00000116783	CANONICAL	singletons	LOF+missense0	168	168	-0,322	0,566	0,570	NA
	ENSG00000116783	CANONICAL	singletons	missense0	142	142	-0,273	0,521	0,601	NA
	ENSG00000116783	CANONICAL	singletons	LOF	26	26	-0,049	0,221	0,824	NA
Supraventricular tachycardia	ENSG00000116783	CANONICAL	MAF<0.1%	LOF+missense0.8	93	2022	8,280	4,629	0,074	NA
	ENSG00000116783	CANONICAL	MAF<0.1%	missense0.8	18	61	0,416	0,760	0,584	NA
	ENSG00000116783	CANONICAL	MAF<0.1%	LOF+missense0.6	172	2589	9,274	5,291	0,080	NA
	ENSG00000116783	CANONICAL	MAF<0.1%	missense0.6	97	628	1,410	2,549	0,580	NA
	ENSG00000116783	CANONICAL	MAF<0.1%	LOF+missense0.4	311	4850	15,796	7,290	0,030	WAAR
	ENSG00000116783	CANONICAL	MAF<0.1%	missense0.4	236	2889	7,933	5,549	0,153	NA
	ENSG00000116783	CANONICAL	MAF<0.1%	LOF+missense0.2	466	6021	17,150	8,102	0,034	WAAR
	ENSG00000116783	CANONICAL	MAF<0.1%	missense0.2	391	4060	9,287	6,578	0,158	NA
	ENSG00000116783	CANONICAL	MAF<0.1%	LOF+missense0	525	6703	15,855	8,537	0,063	NA
	ENSG00000116783	CANONICAL	MAF<0.1%	missense0	450	4742	7,991	7,108	0,261	NA
	ENSG00000116783	CANONICAL	MAF<0.1%	LOF	75	1961	7,864	4,566	0,085	NA
	ENSG00000116783	CANONICAL	MAF<0.001%	LOF+missense0.8	57	114	-0,223	1,122	0,842	NA
	ENSG00000116783	CANONICAL	MAF<0.001%	missense0.8	11	21	0,818	0,424	0,054	NA
	ENSG00000116783	CANONICAL	MAF<0.001%	LOF+missense0.6	107	221	-0,386	1,578	0,807	NA
	ENSG00000116783	CANONICAL	MAF<0.001%	missense0.6	61	128	0,655	1,152	0,570	NA
	ENSG00000116783	CANONICAL	MAF<0.001%	LOF+missense0.4	195	375	-0,008	2,092	0,997	NA
	ENSG00000116783	CANONICAL	MAF<0.001%	missense0.4	149	282	1,033	1,728	0,550	NA
	ENSG00000116783	CANONICAL	MAF<0.001%	LOF+missense0.2	296	569	2,987	2,528	0,237	NA
	ENSG00000116783	CANONICAL	MAF<0.001%	missense0.2	250	476	4,028	2,236	0,072	NA
	ENSG00000116783	CANONICAL	MAF<0.001%	LOF+missense0	326	642	5,259	2,666	0,049	WAAR
	ENSG00000116783	CANONICAL	MAF<0.001%	missense0	280	549	6,300	2,391	0,010	WAAR
	ENSG00000116783	CANONICAL	MAF<0.001%	LOF	46	93	-1,041	1,038	0,316	NA
	ENSG00000116783	CANONICAL	singletons	LOF+missense0.8	32	32	-0,326	0,568	0,566	NA
	ENSG00000116783	CANONICAL	singletons	LOF+missense0.6	55	55	-0,581	0,757	0,443	NA
	ENSG00000116783	CANONICAL	singletons	missense0.6	29	29	-0,308	0,551	0,576	NA
	ENSG00000116783	CANONICAL	singletons	LOF+missense0.4	103	103	-1,056	1,021	0,301	NA
	ENSG00000116783	CANONICAL	singletons	missense0.4	77	77	-0,783	0,879	0,373	NA
	ENSG00000116783	CANONICAL	singletons	LOF+missense0.2	159	159	0,366	1,270	0,773	NA
	ENSG00000116783	CANONICAL	singletons	missense0.2	133	133	0,639	1,159	0,581	NA
	ENSG00000116783	CANONICAL	singletons	LOF+missense0	170	170	1,252	1,314	0,340	NA
	ENSG00000116783	CANONICAL	singletons	missense0	144	144	1,525	1,207	0,206	NA
	ENSG00000116783	CANONICAL	singletons	LOF	26	26	-0,273	0,519	0,599	NA
Supraventricular tachycardia excluding AF	ENSG00000116783	CANONICAL	MAF<0.1%	LOF+missense0.8	88	1708	2,997	2,821	0,288	NA
	ENSG00000116783	CANONICAL	MAF<0.1%	missense0.8	16	54	-0,250	0,499	0,616	NA
	ENSG00000116783	CANONICAL	MAF<0.1%	LOF+missense0.6	163	2173	3,834	3,218	0,234	NA
	ENSG00000116783	CANONICAL	MAF<0.1%	missense0.6	91	519	0,586	1,549	0,705	NA
	ENSG00000116783	CANONICAL	MAF<0.1%	LOF+missense0.4	296	4105	2,657	4,455	0,551	NA
	ENSG00000116783	CANONICAL	MAF<0.1%	missense0.4	224	2451	-0,591	3,402	0,862	NA
	ENSG00000116783	CANONICAL	MAF<0.1%	LOF+missense0.2	444	5119	3,870	4,961	0,435	NA
	ENSG00000116783	CANONICAL	MAF<0.1%	missense0.2	372	3465	0,623	4,040	0,878	NA
	ENSG00000116783	CANONICAL	MAF<0.1%	LOF+missense0	499	5705	3,143	5,229	0,548	NA
	ENSG00000116783	CANONICAL	MAF<0.1%	missense0	427	4051	-0,105	4,366	0,981	NA
	ENSG00000116783	CANONICAL	MAF<0.1%	LOF	72	1654	3,248	2,777	0,242	NA
	ENSG00000116783	CANONICAL	MAF<0.001%	LOF+missense0.8	53	97	-0,479	0,707	0,498	NA
	ENSG00000116783	CANONICAL	MAF<0.001%	LOF+missense0.6	100	186	-0,882	0,962	0,359	NA
	ENSG00000116783	CANONICAL	MAF<0.001%	missense0.6	56	106	-0,486	0,695	0,485	NA
	ENSG00000116783	CANONICAL	MAF<0.001%	LOF+missense0.4	182	319	0,508	1,286	0,693	NA
	ENSG00000116783	CANONICAL	MAF<0.001%	missense0.4	138	239	0,904	1,057	0,392	NA
	ENSG00000116783	CANONICAL	MAF<0.001%	LOF+missense0.2	276	483	0,715	1,566	0,648	NA
	ENSG00000116783	CANONICAL	MAF<0.001%	missense0.2	232	403	1,111	1,384	0,422	NA
	ENSG00000116783	CANONICAL	MAF<0.001%	LOF+missense0	303	543	1,456	1,646	0,376	NA
	ENSG00000116783	CANONICAL	MAF<0.001%	missense0	259	463	1,852	1,474	0,209	NA
	ENSG00000116783	CANONICAL	MAF<0.001%	LOF	44	80	-0,396	0,646	0,540	NA
	ENSG00000116783	CANONICAL	singletons	LOF+missense0.8	31	31	-0,150	0,387	0,698	NA
	ENSG00000116783	CANONICAL	singletons	LOF+missense0.6	58	58	-0,275	0,523	0,599	NA
	ENSG00000116783	CANONICAL	singletons	missense0.6	32	32	-0,148	0,384	0,699	NA
	ENSG00000116783	CANONICAL	singletons	LOF+missense0.4	105	105	-0,475	0,688	0,489	NA
	ENSG00000116783	CANONICAL	singletons	missense0.4	79	79	-0,349	0,589	0,554	NA
	ENSG00000116783	CANONICAL	singletons	LOF+missense0.2	161	161	0,244	0,874	0,780	NA
	ENSG00000116783	CANONICAL	singletons	missense0.2	135	135	0,370	0,798	0,643	NA
	ENSG00000116783	CANONICAL	singletons	LOF+missense0	172	172	0,198	0,899	0,826	NA
	ENSG00000116783	CANONICAL	singletons	missense0	146	146	0,324	0,827	0,695	NA
	ENSG00000116783	CANONICAL	singletons	LOF	26	26	-0,126	0,354	0,722	NA

Ventricular tachycardia	ENSG00000116783	CANONICAL	MAF<0.1%	LOF+missense0.8	93	2022	-0,385	3,203	0,904	NA
	ENSG00000116783	CANONICAL	MAF<0.1%	missense0.8	18	61	0,758	0,490	0,121	NA
	ENSG00000116783	CANONICAL	MAF<0.1%	LOF+missense0.6	172	2589	4,809	3,649	0,188	NA
	ENSG00000116783	CANONICAL	MAF<0.1%	missense0.6	97	628	5,952	1,736	0,002	WAAR
	ENSG00000116783	CANONICAL	MAF<0.1%	LOF+missense0.4	311	4850	6,470	5,001	0,196	NA
	ENSG00000116783	CANONICAL	MAF<0.1%	missense0.4	236	2889	7,613	3,783	0,043	WAAR
	ENSG00000116783	CANONICAL	MAF<0.1%	LOF+missense0.2	466	6021	4,583	5,554	0,409	NA
	ENSG00000116783	CANONICAL	MAF<0.1%	missense0.2	391	4060	5,726	4,488	0,202	NA
	ENSG00000116783	CANONICAL	MAF<0.1%	LOF+missense0	525	6703	3,137	5,854	0,592	NA
	ENSG00000116783	CANONICAL	MAF<0.1%	missense0	450	4742	4,280	4,856	0,378	NA
	ENSG00000116783	CANONICAL	MAF<0.1%	LOF	75	1961	-1,143	3,166	0,718	NA
	ENSG00000116783	CANONICAL	MAF<0.001%	LOF+missense0.8	57	114	0,502	0,713	0,481	NA
	ENSG00000116783	CANONICAL	MAF<0.001%	missense0.8	11	21	0,942	0,239	0,016	WAAR
	ENSG00000116783	CANONICAL	MAF<0.001%	LOF+missense0.6	107	221	0,952	1,058	0,368	NA
	ENSG00000116783	CANONICAL	MAF<0.001%	missense0.6	61	128	1,393	0,775	0,072	NA
	ENSG00000116783	CANONICAL	MAF<0.001%	LOF+missense0.4	195	375	1,196	1,411	0,397	NA
	ENSG00000116783	CANONICAL	MAF<0.001%	missense0.4	149	282	1,637	1,170	0,162	NA
	ENSG00000116783	CANONICAL	MAF<0.001%	LOF+missense0.2	296	569	2,330	1,692	0,169	NA
	ENSG00000116783	CANONICAL	MAF<0.001%	missense0.2	250	476	2,770	1,497	0,064	NA
	ENSG00000116783	CANONICAL	MAF<0.001%	LOF+missense0	326	642	2,985	1,790	0,095	NA
	ENSG00000116783	CANONICAL	MAF<0.001%	missense0	280	549	3,425	1,607	0,030	WAAR
	ENSG00000116783	CANONICAL	MAF<0.001%	LOF	46	93	-0,441	0,671	0,512	NA
	ENSG00000116783	CANONICAL	singletons	LOF+missense0.8	32	32	-0,130	0,359	0,718	NA
	ENSG00000116783	CANONICAL	singletons	LOF+missense0.6	55	55	-0,259	0,506	0,609	NA
	ENSG00000116783	CANONICAL	singletons	missense0.6	29	29	-0,144	0,377	0,703	NA
	ENSG00000116783	CANONICAL	singletons	LOF+missense0.4	103	103	-0,500	0,704	0,477	NA
	ENSG00000116783	CANONICAL	singletons	missense0.4	77	77	-0,385	0,618	0,533	NA
	ENSG00000116783	CANONICAL	singletons	LOF+missense0.2	159	159	0,276	0,847	0,744	NA
	ENSG00000116783	CANONICAL	singletons	missense0.2	133	133	0,391	0,777	0,614	NA
	ENSG00000116783	CANONICAL	singletons	LOF+missense0	170	170	0,217	0,881	0,805	NA
	ENSG00000116783	CANONICAL	singletons	missense0	144	144	0,332	0,813	0,683	NA
	ENSG00000116783	CANONICAL	singletons	LOF	26	26	-0,115	0,338	0,733	NA

Note: abbreviations: cMAC, cumulative minor allele count; SE, standard error; SPA, saddle-point approximation; MAF, minor allele count; LOF, loss-of-function; NA, not applicable

Table IX. Cauchy association results for rare TNNI3K variants and diseases in UK Biobank

Phenotype	Transcript	Variants	Cauchy *P*-value
Original result testing in individuals of all ancestry			
Atrial fibrillation or flutter (AF)	CANONICAL	LOF	0,189
	CANONICAL	missense	0,030
	CANONICAL	LOF+missense	0,018
	**CANONICAL**	**COMBINED**	**0,032**
Conduction system disease	CANONICAL	LOF	0,500
	CANONICAL	missense	0,410
	CANONICAL	LOF+missense	0,836
	**CANONICAL**	**COMBINED**	**0,646**
Hypertrophic cardiomyopathy	CANONICAL	LOF	0,497
	CANONICAL	missense	0,022
	CANONICAL	LOF+missense	0,036
	**CANONICAL**	**COMBINED**	**0,040**
Dilated cardiomyopathy	CANONICAL	LOF	0,408
	CANONICAL	missense	0,001
	CANONICAL	LOF+missense	0,027
	**CANONICAL**	**COMBINED**	**0,003**
Supraventricular tachycardia	CANONICAL	LOF	0,247
	CANONICAL	missense	0,141
	CANONICAL	LOF+missense	0,109
	**CANONICAL**	**COMBINED**	**0,149**
Ventricular tachycardia	CANONICAL	LOF	0,761
	CANONICAL	missense	0,055
	CANONICAL	LOF+missense	0,881
	**CANONICAL**	**COMBINED**	**0,305**
Sensitivity analysis testing only in individuals of European ancestry			
**Phenotype**	**Transcript**	**Variants**	**Cauchy *P*-value**
Atrial fibrillation or flutter (AF)	CANONICAL	LOF	0,184
	CANONICAL	missense	0,029
	CANONICAL	LOF+missense	0,017
	**CANONICAL**	**COMBINED**	**0,031**
Conduction system disease	CANONICAL	LOF	0,367
	CANONICAL	missense	0,957
	CANONICAL	LOF+missense	0,874
	**CANONICAL**	**COMBINED**	**0,901**
Hypertrophic cardiomyopathy	CANONICAL	LOF	0,620
	CANONICAL	missense	0,346
	CANONICAL	LOF+missense	0,223
	**CANONICAL**	**COMBINED**	**0,369**
Dilated cardiomyopathy	CANONICAL	LOF	0,484
	CANONICAL	missense	0,002
	CANONICAL	LOF+missense	0,036
	**CANONICAL**	**COMBINED**	**0,006**
Supraventricular tachycardia	CANONICAL	LOF	0,205
	CANONICAL	missense	0,090
	CANONICAL	LOF+missense	0,934
	**CANONICAL**	**COMBINED**	**0,494**
Ventricular tachycardia	CANONICAL	LOF	0,668
	CANONICAL	missense	0,023
	CANONICAL	LOF+missense	0,458
	**CANONICAL**	**COMBINED**	**0,071**

Note: abbreviations: LOF, loss-of-function

Table X. Frequency of rare TNNI3K variants among disease cases and controls in UK Biobank

All ancestry

Phenotype	Variant Mask	Carriers among cases, N	Total cases, N	Carriers among cases, %	Carriers among controls, N	Total controls	Carriers among controls, %	log(OR)	SE of log(OR)	OR	95%CI of OR
Atrial fibrillation or flutter	LOF+missense0.8 (MAF<0.1%)	151	29149	0,52	1969	425013	0,46	0,12	0,09	1,12	[0.95; 1.34]
	missense0.8 (MAF<0.1%)	3	29149	0,01	71	425013	0,02	0,11	0,57	1,12	[0.37; 3.39]
	LOF+missense0.6 (MAF<0.1%)	195	29149	0,67	2521	425013	0,59	0,14	0,08	1,16	[0.99; 1.35]
	missense0.6 (MAF<0.1%)	51	29149	0,18	651	425013	0,15	0,27	0,15	1,31	[0.97; 1.77]
	LOF+missense0.4 (MAF<0.1%)	367	29149	1,26	4649	425013	1,09	0,15	0,06	1,17	[1.04; 1.31]
	missense0.4 (MAF<0.1%)	229	29149	0,79	2820	425013	0,66	0,20	0,07	1,22	[1.06; 1.4]
	LOF+missense0.2 (MAF<0.1%)	459	29149	1,58	5899	425013	1,39	0,15	0,05	1,16	[1.05; 1.29]
	missense0.2 (MAF<0.1%)	322	29149	1,11	4076	425013	0,96	0,18	0,06	1,20	[1.06; 1.35]
	LOF+missense0 (MAF<0.1%)	495	29149	1,70	6586	425013	1,55	0,12	0,05	1,12	[1.02; 1.24]
	missense0 (MAF<0.1%)	358	29149	1,23	4764	425013	1,12	0,13	0,06	1,14	[1.02; 1.28]
	LOF (MAF<0.1%)	148	29149	0,51	1898	425013	0,45	0,12	0,09	1,13	[0.95; 1.34]
Conduction system disease	LOF+missense0.8 (MAF<0.1%)	54	12331	0,44	2066	441831	0,47	-0,04	0,14	0,96	[0.73; 1.26]
	missense0.8 (MAF<0.1%)	0	12331	0,00	74	441831	0,02	-1,03	1,43	0,36	[0.02; 5.86]
	LOF+missense0.6 (MAF<0.1%)	74	12331	0,60	2642	441831	0,60	0,03	0,12	1,03	[0.81; 1.31]
	missense0.6 (MAF<0.1%)	22	12331	0,18	680	441831	0,15	0,25	0,23	1,29	[0.82; 2.01]
	LOF+missense0.4 (MAF<0.1%)	151	12331	1,23	4865	441831	1,10	0,13	0,09	1,14	[0.97; 1.35]
	missense0.4 (MAF<0.1%)	99	12331	0,80	2950	441831	0,67	0,23	0,11	1,25	[1.02; 1.54]
	LOF+missense0.2 (MAF<0.1%)	185	12331	1,50	6173	441831	1,40	0,09	0,08	1,10	[0.94; 1.28]
	missense0.2 (MAF<0.1%)	134	12331	1,09	4264	441831	0,97	0,15	0,09	1,16	[0.97; 1.39]
	LOF+missense0 (MAF<0.1%)	198	12331	1,61	6883	441831	1,56	0,05	0,08	1,05	[0.91; 1.22]
	missense0 (MAF<0.1%)	147	12331	1,19	4975	441831	1,13	0,09	0,09	1,09	[0.92; 1.3]
	LOF (MAF<0.1%)	54	12331	0,44	1992	441831	0,45	-0,02	0,14	0,98	[0.75; 1.29]
Hypertrophic cardiomyopathy	LOF+missense0.8 (MAF<0.1%)	0	538	0,00	2120	453624	0,47	-1,51	1,41	0,22	[0.01; 3.49]
	missense0.8 (MAF<0.1%)	0	538	0,00	74	453624	0,02	2,07	1,41	7,90	[0.49; 126]
	LOF+missense0.6 (MAF<0.1%)	2	538	0,37	2714	453624	0,60	-0,64	0,81	0,53	[0.11; 2.6]
	missense0.6 (MAF<0.1%)	2	538	0,37	700	453624	0,15	0,80	0,81	2,22	[0.45; 10.95]
	LOF+missense0.4 (MAF<0.1%)	4	538	0,74	5012	453624	1,11	-0,41	0,53	0,67	[0.23; 1.9]
	missense0.4 (MAF<0.1%)	4	538	0,74	3045	453624	0,67	0,11	0,53	1,12	[0.39; 3.2]
	LOF+missense0.2 (MAF<0.1%)	5	538	0,93	6353	453624	1,40	-0,38	0,47	0,69	[0.27; 1.73]
	missense0.2 (MAF<0.1%)	5	538	0,93	4393	453624	0,97	0,02	0,47	1,02	[0.4; 2.57]
	LOF+missense0 (MAF<0.1%)	5	538	0,93	7076	453624	1,56	-0,48	0,47	0,62	[0.24; 1.55]
	missense0 (MAF<0.1%)	5	538	0,93	5117	453624	1,13	-0,14	0,47	0,87	[0.35; 2.19]
	LOF (MAF<0.1%)	0	538	0,00	2046	453624	0,45	-1,48	1,41	0,23	[0.01; 3.59]
Dilated cardiomyopathy	LOF+missense0.8 (MAF<0.1%)	1	924	0,11	2051	438722	0,47	-1,04	0,82	0,35	[0.07; 1.75]
	missense0.8 (MAF<0.1%)	0	924	0,00	71	438722	0,02	1,60	1,42	4,95	[0.31; 80.16]
	LOF+missense0.6 (MAF<0.1%)	7	924	0,76	2624	438722	0,60	0,22	0,39	1,24	[0.57; 2.68]
	missense0.6 (MAF<0.1%)	6	924	0,65	675	438722	0,15	1,53	0,43	4,60	[1.98; 10.67]
	LOF+missense0.4 (MAF<0.1%)	18	924	1,95	4844	438722	1,10	0,53	0,25	1,71	[1.05; 2.78]
	missense0.4 (MAF<0.1%)	17	924	1,84	2937	438722	0,67	1,00	0,26	2,71	[1.64; 4.48]
	LOF+missense0.2 (MAF<0.1%)	22	924	2,38	6128	438722	1,40	0,54	0,22	1,72	[1.11; 2.66]
	missense0.2 (MAF<0.1%)	21	924	2,27	4228	438722	0,96	0,89	0,23	2,44	[1.55; 3.82]
	LOF+missense0 (MAF<0.1%)	24	924	2,60	6826	438722	1,56	0,53	0,21	1,70	[1.12; 2.58]
	missense0 (MAF<0.1%)	23	924	2,49	4927	438722	1,12	0,83	0,22	2,30	[1.5; 3.53]
	LOF (MAF<0.1%)	1	924	0,11	1980	438722	0,45	-1,01	0,82	0,36	[0.07; 1.8]
Ventricular tachycardia	LOF+missense0.8 (MAF<0.1%)	11	2297	0,48	2109	451865	0,47	0,01	0,31	1,01	[0.55; 1.86]
	missense0.8 (MAF<0.1%)	1	2297	0,04	73	451865	0,02	1,84	0,83	6,30	[1.23; 32.26]
	LOF+missense0.6 (MAF<0.1%)	19	2297	0,83	2697	451865	0,60	0,36	0,23	1,43	[0.9; 2.26]
	missense0.6 (MAF<0.1%)	9	2297	0,39	693	451865	0,15	1,16	0,33	3,18	[1.67; 6.06]
	LOF+missense0.4 (MAF<0.1%)	33	2297	1,44	4983	451865	1,10	0,28	0,18	1,32	[0.93; 1.88]
	missense0.4 (MAF<0.1%)	23	2297	1,00	3026	451865	0,67	0,46	0,21	1,59	[1.04; 2.41]
	LOF+missense0.2 (MAF<0.1%)	37	2297	1,61	6321	451865	1,40	0,18	0,17	1,19	[0.86; 1.67]
	missense0.2 (MAF<0.1%)	27	2297	1,18	4371	451865	0,97	0,28	0,20	1,32	[0.9; 1.94]
	LOF+missense0 (MAF<0.1%)	39	2297	1,70	7042	451865	1,56	0,12	0,17	1,13	[0.82; 1.56]
	missense0 (MAF<0.1%)	29	2297	1,26	5093	451865	1,13	0,19	0,19	1,21	[0.84; 1.76]
	LOF (MAF<0.1%)	10	2297	0,44	2036	451865	0,45	-0,07	0,33	0,94	[0.49; 1.77]
Supraventricular tachycardia	LOF+missense0.8 (MAF<0.1%)	30	4836	0,62	2090	449326	0,47	0,35	0,18	1,41	[0.99; 2.02]
	missense0.8 (MAF<0.1%)	1	4836	0,02	73	449326	0,02	0,95	0,83	2,58	[0.51; 13.05]
	LOF+missense0.6 (MAF<0.1%)	39	4836	0,81	2677	449326	0,60	0,32	0,17	1,38	[1; 1.91]
	missense0.6 (MAF<0.1%)	10	4836	0,21	692	449326	0,15	0,26	0,35	1,30	[0.66; 2.55]
	LOF+missense0.4 (MAF<0.1%)	70	4836	1,45	4946	449326	1,10	0,27	0,12	1,31	[1.03; 1.67]
	missense0.4 (MAF<0.1%)	42	4836	0,87	3007	449326	0,67	0,22	0,16	1,25	[0.91; 1.72]
	LOF+missense0.2 (MAF<0.1%)	83	4836	1,72	6275	449326	1,40	0,22	0,11	1,24	[0.99; 1.55]
	missense0.2 (MAF<0.1%)	56	4836	1,16	4342	449326	0,97	0,17	0,14	1,19	[0.9; 1.57]
	LOF+missense0 (MAF<0.1%)	89	4836	1,84	6992	449326	1,56	0,18	0,11	1,20	[0.97; 1.49]
	missense0 (MAF<0.1%)	62	4836	1,28	5060	449326	1,13	0,13	0,13	1,14	[0.88; 1.48]
	LOF (MAF<0.1%)	29	4836	0,60	2017	449326	0,45	0,34	0,19	1,40	[0.97; 2.02]
Suparaventricular tachycardia excluding AF	LOF+missense0.8 (MAF<0.1%)	13	2111	0,62	2090	449326	0,47	0,38	0,27	1,46	[0.85; 2.49]
	missense0.8 (MAF<0.1%)	0	2111	0,00	73	449326	0,02	0,59	1,42	1,81	[0.11; 29.23]
	LOF+missense0.6 (MAF<0.1%)	17	2111	0,81	2677	449326	0,60	0,34	0,25	1,41	[0.87; 2.29]
	missense0.6 (MAF<0.1%)	4	2111	0,19	692	449326	0,15	0,19	0,54	1,21	[0.42; 3.46]
	LOF+missense0.4 (MAF<0.1%)	28	2111	1,33	4946	449326	1,10	0,19	0,20	1,20	[0.82; 1.77]
	missense0.4 (MAF<0.1%)	16	2111	0,76	3007	449326	0,67	0,08	0,26	1,08	[0.64; 1.81]
	LOF+missense0.2 (MAF<0.1%)	34	2111	1,61	6275	449326	1,40	0,17	0,18	1,19	[0.84; 1.68]
	missense0.2 (MAF<0.1%)	22	2111	1,04	4342	449326	0,97	0,09	0,22	1,09	[0.7; 1.68]
	LOF+missense0 (MAF<0.1%)	36	2111	1,71	6992	449326	1,56	0,12	0,17	1,13	[0.81; 1.58]
	missense0 (MAF<0.1%)	24	2111	1,14	5060	449326	1,13	0,03	0,21	1,03	[0.68; 1.55]
	LOF (MAF<0.1%)	13	2111	0,62	2017	449326	0,45	0,41	0,27	1,50	[0.88; 2.57]

Note: abbreviations: LOF, loss-of-function

European ancestry sensitivity analysis

Phenotype	Variant Mask	Carriers among cases, N	Cases, N	Carriers among cases, %	Carriers among controls, N	Controls, N	Carriers among controls, %	log(OR)	SE of log(OR)	OR	95%CI of OR
Atrial fibrillation or flutter	LOF+missense0.8 (MAF<0.1%)	151	28339	0,522	1870	400533	0,467	0,11	0,09	1,12	[0.94; 1.33]
	missense0.8 (MAF<0.1%)	3	28339	0,011	58	400533	0,014	-0,05	0,56	0,96	[0.32; 2.87]
	LOF+missense0.6 (MAF<0.1%)	195	28339	0,67	2363	400533	0,59	0,14	0,08	1,15	[0.99; 1.33]
	missense0.6 (MAF<0.1%)	51	28339	0,173	579	400533	0,145	0,24	0,15	1,27	[0.95; 1.7]
	LOF+missense0.4 (MAF<0.1%)	367	28339	1,263	4396	400533	1,098	0,15	0,06	1,17	[1.04; 1.3]
	missense0.4 (MAF<0.1%)	229	28339	0,787	2652	400533	0,662	0,20	0,07	1,22	[1.06; 1.4]
	LOF+missense0.2 (MAF<0.1%)	459	28339	1,563	5466	400533	1,365	0,15	0,05	1,16	[1.05; 1.28]
	missense0.2 (MAF<0.1%)	322	28339	1,09	3727	400533	0,931	0,18	0,06	1,20	[1.06; 1.35]
	LOF+missense0 (MAF<0.1%)	495	28339	1,687	6104	400533	1,524	0,11	0,05	1,12	[1.02; 1.23]
	missense0 (MAF<0.1%)	358	28339	1,214	4366	400533	1,09	0,13	0,06	1,13	[1.01; 1.27]
	LOF (MAF<0.1%)	148	28339	0,512	1812	400533	0,452	0,12	0,09	1,13	[0.95; 1.34]
Conduction system disease	LOF+missense0.8 (MAF<0.1%)	53	11758	0,451	1965	417114	0,471	-0,04	0,14	0,96	[0.73; 1.26]
	missense0.8 (MAF<0.1%)	0	11758	0	61	417114	0,015	-1,03	1,43	0,36	[0.02; 5.86]
	LOF+missense0.6 (MAF<0.1%)	71	11758	0,604	2482	417114	0,595	0,03	0,12	1,03	[0.81; 1.31]
	missense0.6 (MAF<0.1%)	20	11758	0,17	608	417114	0,146	0,25	0,23	1,29	[0.82; 2.01]
	LOF+missense0.4 (MAF<0.1%)	147	11758	1,25	4607	417114	1,104	0,13	0,09	1,14	[0.97; 1.35]
	missense0.4 (MAF<0.1%)	96	11758	0,816	2779	417114	0,666	0,23	0,11	1,25	[1.02; 1.54]
	LOF+missense0.2 (MAF<0.1%)	176	11758	1,497	5733	417114	1,374	0,09	0,08	1,10	[0.94; 1.28]
	missense0.2 (MAF<0.1%)	126	11758	1,072	3910	417114	0,937	0,15	0,09	1,16	[0.97; 1.39]
	LOF+missense0 (MAF<0.1%)	188	11758	1,599	6394	417114	1,533	0,05	0,08	1,05	[0.91; 1.22]
	missense0 (MAF<0.1%)	138	11758	1,174	4572	417114	1,096	0,09	0,09	1,09	[0.92; 1.3]
	LOF (MAF<0.1%)	53	11758	0,451	1904	417114	0,456	-0,02	0,14	0,98	[0.75; 1.29]
Hypertrophic cardiomyopathy	LOF+missense0.8 (MAF<0.1%)	0	482	0	2018	428390	0,471	-1,63	1,41	0,20	[0.01; 3.12]
	missense0.8 (MAF<0.1%)	0	482	0	61	428390	0,014	1,81	1,41	6,12	[0.39; 96.95]
	LOF+missense0.6 (MAF<0.1%)	1	482	0,207	2552	428390	0,596	-0,27	0,63	0,77	[0.22; 2.64]
	missense0.6 (MAF<0.1%)	1	482	0,207	627	428390	0,146	1,09	0,63	2,97	[0.86; 10.26]
	LOF+missense0.4 (MAF<0.1%)	3	482	0,622	4751	428390	1,109	-0,27	0,47	0,77	[0.3; 1.93]
	missense0.4 (MAF<0.1%)	3	482	0,622	2872	428390	0,67	0,25	0,47	1,29	[0.51; 3.25]
	LOF+missense0.2 (MAF<0.1%)	4	482	0,83	5905	428390	1,378	-0,33	0,43	0,72	[0.31; 1.66]
	missense0.2 (MAF<0.1%)	4	482	0,83	4032	428390	0,941	0,04	0,43	1,04	[0.45; 2.4]
	LOF+missense0 (MAF<0.1%)	4	482	0,83	6578	428390	1,536	-0,44	0,43	0,64	[0.28; 1.48]
	missense0 (MAF<0.1%)	4	482	0,83	4706	428390	1,099	-0,11	0,43	0,89	[0.39; 2.06]
	LOF (MAF<0.1%)	0	482	0	1957	428390	0,457	-1,59	1,41	0,20	[0.01; 3.22]
Dilated cardiomyopathy	LOF+missense0.8 (MAF<0.1%)	1	869	0,115	1952	414294	0,471	-1,09	0,82	0,34	[0.07; 1.67]
	missense0.8 (MAF<0.1%)	0	869	0	58	414294	0,014	1,37	1,41	3,95	[0.25; 63.02]
	LOF+missense0.6 (MAF<0.1%)	6	869	0,69	2468	414294	0,596	0,29	0,37	1,34	[0.65; 2.74]
	missense0.6 (MAF<0.1%)	5	869	0,575	605	414294	0,146	1,56	0,40	4,74	[2.18; 10.27]
	LOF+missense0.4 (MAF<0.1%)	16	869	1,841	4594	414294	1,109	0,59	0,23	1,81	[1.14; 2.87]
	missense0.4 (MAF<0.1%)	15	869	1,726	2772	414294	0,669	1,06	0,24	2,88	[1.79; 4.63]
	LOF+missense0.2 (MAF<0.1%)	20	869	2,301	5697	414294	1,375	0,55	0,21	1,74	[1.14; 2.64]
	missense0.2 (MAF<0.1%)	19	869	2,186	3881	414294	0,937	0,89	0,22	2,44	[1.59; 3.74]
	LOF+missense0 (MAF<0.1%)	22	869	2,532	6347	414294	1,532	0,53	0,20	1,70	[1.14; 2.54]
	missense0 (MAF<0.1%)	21	869	2,417	4532	414294	1,094	0,83	0,21	2,28	[1.52; 3.44]
	LOF (MAF<0.1%)	1	869	0,115	1894	414294	0,457	-1,06	0,82	0,35	[0.07; 1.71]
Ventricular tachycardia	LOF+missense0.8 (MAF<0.1%)	10	2172	0,46	2008	426700	0,471	0,06	0,30	1,07	[0.6; 1.91]
	missense0.8 (MAF<0.1%)	1	2172	0,046	60	426700	0,014	1,58	0,83	4,86	[0.95; 24.75]
	LOF+missense0.6 (MAF<0.1%)	18	2172	0,829	2535	426700	0,594	0,36	0,23	1,43	[0.91; 2.23]
	missense0.6 (MAF<0.1%)	9	2172	0,414	619	426700	0,145	1,03	0,33	2,81	[1.48; 5.35]
	LOF+missense0.4 (MAF<0.1%)	31	2172	1,427	4723	426700	1,107	0,29	0,17	1,34	[0.95; 1.89]
	missense0.4 (MAF<0.1%)	22	2172	1,013	2853	426700	0,669	0,45	0,21	1,57	[1.04; 2.36]
	LOF+missense0.2 (MAF<0.1%)	35	2172	1,611	5874	426700	1,377	0,16	0,17	1,18	[0.85; 1.63]
	missense0.2 (MAF<0.1%)	26	2172	1,197	4010	426700	0,94	0,23	0,19	1,26	[0.86; 1.84]
	LOF+missense0 (MAF<0.1%)	37	2172	1,703	6545	426700	1,534	0,11	0,16	1,11	[0.81; 1.53]
	missense0 (MAF<0.1%)	28	2172	1,289	4682	426700	1,097	0,14	0,19	1,16	[0.8; 1.66]
	LOF (MAF<0.1%)	9	2172	0,414	1948	426700	0,457	0,00	0,31	1,00	[0.55; 1.84]
Supraventricular tachycardia	LOF+missense0.8 (MAF<0.1%)	30	4631	0,648	1988	424241	0,469	0,31	0,18	1,36	[0.95; 1.95]
	missense0.8 (MAF<0.1%)	1	4631	0,022	60	424241	0,014	0,79	0,83	2,20	[0.44; 11.09]
	LOF+missense0.6 (MAF<0.1%)	37	4631	0,799	2516	424241	0,593	0,33	0,16	1,39	[1.01; 1.91]
	missense0.6 (MAF<0.1%)	8	4631	0,173	620	424241	0,146	0,39	0,31	1,47	[0.8; 2.71]
	LOF+missense0.4 (MAF<0.1%)	66	4631	1,425	4688	424241	1,105	0,29	0,12	1,34	[1.05; 1.69]
	missense0.4 (MAF<0.1%)	38	4631	0,821	2837	424241	0,669	0,28	0,16	1,33	[0.98; 1.8]
	LOF+missense0.2 (MAF<0.1%)	78	4631	1,684	5831	424241	1,374	0,22	0,11	1,25	[1.01; 1.56]
	missense0.2 (MAF<0.1%)	51	4631	1,101	3985	424241	0,939	0,20	0,13	1,23	[0.94; 1.6]
	LOF+missense0 (MAF<0.1%)	84	4631	1,814	6498	424241	1,532	0,19	0,11	1,20	[0.98; 1.49]
	missense0 (MAF<0.1%)	57	4631	1,231	4653	424241	1,097	0,15	0,13	1,16	[0.9; 1.5]
	LOF (MAF<0.1%)	29	4631	0,626	1928	424241	0,454	0,31	0,19	1,36	[0.94; 1.96]
Suparaventricular tachycardia excluding AF	LOF+missense0.8 (MAF<0.1%)	13	1993	0,652	1988	424241	0,469	0,33	0,27	1,39	[0.81; 2.38]
	missense0.8 (MAF<0.1%)	0	1993	0	60	424241	0,014	0,42	1,42	1,52	[0.09; 24.47]
	LOF+missense0.6 (MAF<0.1%)	16	1993	0,803	2516	424241	0,593	0,34	0,24	1,41	[0.88; 2.25]
	missense0.6 (MAF<0.1%)	3	1993	0,151	620	424241	0,146	0,33	0,47	1,40	[0.55; 3.53]
	LOF+missense0.4 (MAF<0.1%)	26	1993	1,305	4688	424241	1,105	0,21	0,19	1,23	[0.85; 1.78]
	missense0.4 (MAF<0.1%)	14	1993	0,702	2837	424241	0,669	0,15	0,25	1,17	[0.72; 1.89]
	LOF+missense0.2 (MAF<0.1%)	32	1993	1,606	5831	424241	1,374	0,16	0,17	1,17	[0.84; 1.65]
	missense0.2 (MAF<0.1%)	20	1993	1,004	3985	424241	0,939	0,10	0,21	1,10	[0.73; 1.67]
	LOF+missense0 (MAF<0.1%)	34	1993	1,706	6498	424241	1,532	0,11	0,17	1,12	[0.8; 1.55]
	missense0 (MAF<0.1%)	22	1993	1,104	4653	424241	1,097	0,03	0,20	1,03	[0.69; 1.53]
	LOF (MAF<0.1%)	13	1993	0,652	1928	424241	0,454	0,36	0,27	1,44	[0.84; 2.46]

Note: Effect sizes estimated from Firth's regression models among unrelated samples. Abbrevations: LOF, loss-of-function

Table XI. Clinical characteristics of TNNI3K-p.Ile512Thr and TNNI3K-p.His592Tyr families

General						Symptoms	Arrhythmias							Exercise test	Holter	Latest ECG							Echocardiography at admission				MRI				Life-threatening events	Hypertension	Therapy			
ID	M/F	Age	Variant carrier	Other variants	Age of diagnosis	Syncope and palpitations	SVT	AVNRT	AF	Atrial flutter	JET	VT	Other			HR (bpm)	PR interval (ms)	LBBB	RBBB	AVB	LAD	Other	General	LVEF	LVEDD (mm)	Others	General comments	LVEF	LVEDD	Fibrosis			Current drug therapy	Pacemaker	ICD	Ablation
**TNNI3K-p.Ile512Thr**																																				
IV-4	M	24	+/-	+/- (*LAMA4**)	childhood	Syncope	-	-	-	-	-	-	Negative Ajmaline test	ND	ND	ND	ND	-	+	-	+	LAFB	Normal	ND	ND	-	ND	ND	ND	ND	-	-	-	-	-	-
IV-5	M	26	+/-	-	ND	-	-	-	-	-	-	-	-	ND	ND	ND	ND	-	+	-	-	RAD	Normal	ND	ND	-	ND	ND	ND	ND	-	-	-	-	-	-
II-3	F	75	+/-	-	65-70	-	+	-	+	-	-	-	-	ND	ND	ND	ND	+	-	-	-	-	DCM	ND	ND	-	ND	ND	ND	ND	-	-	ND	CRT-D	-	-
III-3	F	54	+/-	+/- (*LAMA4**)	childhood	Syncope	ND	ND	ND	ND	ND	ND	-	ND	ND	ND	ND	+	-	-	+	-	DCM – dilated RV	51%	ND	-	ND	ND	ND	ND	-	ND	ND	-	-	-
**TNNI3K-p.H592T**																																				
1.II-4	M	70	+/-	-	50-55	Reduced exercise tolerance	+	-	-	-	-	-	Short series of NSVT	ND	ND	63	220	+	-	1st degree	+	Abnormal ST-segments	DCM (LV)	20%	56-57	3rd grade MI	ND	ND	ND	ND	-	-	Acenocoumarin, amitriptyline, carvedilol, furosemide, rosuvastatin, valsartan	CRT-D	-	-
1.III-2	F	48	+/-	-	30-35	Palpitations, reduced exercise tolerance	-	-	-	-	-	-	-	ND	ND	93	ND	-	-	-	+	-	Mild DCM, round morphology of the heart and chambers	45%	ND	-	Mild reduced LV function	50-63%	Normal	-	-	-	Candesartan	-	-	-
1.III-3	M	46	-/-	-	ND	-	-	-	-	-	-	-	-	Normal	Normal	56	ND	-	-	-	-	Normal ECG	Normal morphology	45%	49	1st grade MI	LVEF=50%	ND	ND	ND	-	-	Candesartan	-	-	-
1.III-4	F	41	+/-	-	30-35	Reduced exercise tolerance	-	-	-	-	-	-	-	Normal	Normal	ND	ND	-	-	-	-	Normal ECG	Borderline dimensions of LV and RV	decreased	56	1st grade MI	Reduced LV function	46%	55	-	-	-	Ramipril	-	-	-
2.II-1	F	68	+/-	-	50-55	-	ND	ND	ND	ND	ND	+	-	ND	ND	60	200	+	-	1st degree	-	-	DCM (LV)	25%	ND	desynchrony	-	43%	ND	ND	OHCA	ND	ND	-	+	-
2.I-3	F	ND	+/-	-	60-65	-	ND	ND	ND	ND	ND	ND	-	ND	ND	ND	ND	ND	ND	3rd degree	ND	-	ND	ND	ND	-	ND	ND	ND	ND	ND	ND	ND	CTR	-	ND
3.II-1	F	34	+/-	-	25-30	Syncope, palpitations, chest pain	+	+	-	+	+	Possible	PVCs, VES, ventricular escape rhythm, tachycardia with LBBB morphology, possible VTs/SVTs	Tachycardia with LBBB morphology during exercise, possible VTs/SVTs/ atypical AVNRT	Frequent symptomatic	86	176	-	-	+	-	RAD	Normal	ND	ND	-	-	60%	ND	-	Decompensation during arrhythmic event	-	-	-	-	x2
3.I-2	F	60	-/-	-	Healthy	Syncope	-	-	-	-	-	-	-	ND	ND	ND	ND	ND	ND	ND	ND	-	Normal	ND	ND	-	ND	ND	ND	ND	-	ND	ND	-	-	-
4.II-2	F	52	+/-	+/- (*RBM20***)	30-35	Palpitations, reduced exercise tolerance, chest pain, dyspnoea	+	+	-	-	+	-	Incessant AV synchrony post-ablation, possible junctional tachycardia	Typical AVNRT	Junctional tachycardia	70	174	-	-	-	-	Earlier history of sinus tachycardia with RAD	Peripartum cardiomyopathy, currently mild DCM	28% (Now 47%)	69 (Now 58)	Partial recovery of LV function	ND	ND	ND	ND	-	+	-	-	-	x2
4.III-1	F	30	+/-	-	Healthy	-	-	-	-	-	-	-	-	ND	ND	80	-	-	-	-	-	Normal ECG	Normal	ND	ND	-	ND	ND	ND	ND	-	+	Metoprolol	-	-	-
4.III-2	F	19	-/-	-	Healthy	-	-	-	-	-	-	-	-	ND	ND	67	-	-	-	-	-	Normal ECG	Normal	ND	ND	-	ND	ND	ND	ND	-	-	-	-	-	-
5.II-2	F	68	+/-	-	55-60	Syncope, palpitations	+	+	+	-	-	-	PVCs, atrial tachycardia, broad complex tachycardia with aberration of LBBB morphology	ND	ND	77	254	+	-	+	-	-	Mild concentric remodelling	40%	ND	2nd grade MI	DCM	36%	62	Mid-wall	Decompensation during arrhythmic event	+	Apixaban, metoprolol, sacubitril/valsartan, spironolactone, temazepam	-	-	Multiple pharmaco-logical and catheter ablations
5.III-2	F	41	+/-	-	Healthy	-	-	-	-	-	-	-	-	ND	ND	ND	ND	ND	ND	ND	ND	ND	ND	ND	ND	ND	ND	ND	ND	ND	ND	ND	ND	ND	ND	ND
6.IV-1	M	75	-/-	-	Healthy	-	-	-	-	-	-	-	-	ND	ND	ND	ND	ND	ND	ND	ND	ND	ND	ND	ND	ND	ND	ND	ND	ND	-	-	-	-	-	-
6.IV-2	F	79	+/-	-	40-43	Palpitations, angina pectoris	-	-	+	-	-	+	nVT	ND	ND	ND	-	+	-	-	-	-	Dilated LA	15-25%	ND	ND	ND	ND	ND	ND	-	-	-	-	+	-
6.V-1	M	56	+/-	-	50-55	-	ND	ND	+	ND	ND	ND	SCD	ND	ND	ND	ND	-	-	-	-	-	DCM (LV)	ND	ND	ND	ND	ND	ND	ND	SCD	-	-	-	-	-
6.V-2	M	50	+/-	-	45-50	-	-	-	-	-	-	+	-	VT	ND	ND	ND	ND	ND	ND	ND	ND	ND	ND	ND	ND	ND	ND	ND	ND	-	-	-	-	+	-

* LAMA4 c.2555C>T; p.(Thr852Met) Class 3
** RBM20-c.3115C>T; p.(Pro1039Ser) Class 3
ND: No data
AVNRT – atrio-ventricular nodal reentry tachycardia; AVB – atrioventricular block; AF – atrial fibrillation; CJET – congenital junctional ectopic tachycardia; CPVT – catecholaminergic polymorphic ventricular tachycardia; DCM – dilated cardiomyopathy; JET – junction ectopic tachycardia; LBBB – left bundle branch block; LAD – left axis deviation; LAFB - left anterior fascicular block; LVEDD – left ventricular end-diastolic dimension; LVEF – left ventricular ejection fraction; MI – myocardial infarction; nVT – non-sustained ventricular tachycardia; OHCA – out-of-hospital cardiac arrest; PVCs – premature cardiac contractions; RAD – right axis deviation; RBBB – right bundle branch block; RV – right ventricle; SCD – sudden cardiac death; SSS – sick sinus syndrome; SVT – supraventricular tachycardia; VT – ventricular tachycardia

Supplemental Methods

Genetic screening and enrichment analysis

Data were collected from patients referred for genetic examination of the cardiomyopathy and arrhythmia panels from June 2018 to December 2021. *TNNI3K* (NM_015978.2) was included in both panels in June 2018 and was present for the whole period of genetic screening. The cardiomyopathy panel consists of 56 genes and no major changes were introduced during the screening period. The arrhythmia panel included 54 or 60 genes (Supplemental Table SI). Patients who underwent genetic screening in Amsterdam University Medical Center, Amsterdam were referred by six centers across the Netherlands: (I) Amsterdam University Medical Center, Amsterdam; (II) Leiden University Medical Center, Leiden; (III) Erasmus Medical Center, Rotterdam; (IV) University Medical Center, Groningen, (V) University Medical Center Utrecht, Utrecht, (VI) Groene Hart Ziekenhuis, Gouda, and (VII) Medisch Spectrum Twente, Enschede. Other patients with reported *TNNI3K* variants consisted of family members who underwent selective examination of the variant to determine the familial segregation or genetic screening of the panels established and performed in previously mentioned institutions.

The Genome Aggregation Database (gnomAD) is a population-based data set, which was used as our control cohort[16]. The v2.1.1 data set consists of 125,748 exome sequences and 15,708 whole-genome sequences of unrelated individuals from different ancestries. The expanded v3.1.2 database comprises 76,156 genomes.

We refined the list of screened *TNNI3K* variants by excluding: (I) common *TNNI3K* variants, with minor allele frequency (MAF) >0.0001 in gnomAD (v3.1.2: ENST00000326637.8/NM_015978.3) and (II) variants that were not predicted to alter the encoded protein (i.e. variants in the 5’ and 3’ untranslated regions, synonymous, intronic, and non-splice related variants). For the enrichment analysis, we used the exome data from gnomAD v2.1.1 with the *TNNI3K* reference transcript: ENST00000326637.3. The exclusion criteria for variants in *TNNI3K* in both the Amsterdam UMC cohort and the control cohort

consisted of variants with MAF>0.0001, synonymous variants, intronic variants, and variants in the 5' and 3' untranslated regions. The frequency of the variants was statistically compared as described below.

TNNI3K disease associations in the UK Biobank

The UK Biobank is a prospective cohort study that included over 500,000 individuals of middle age (40-69 at enrollment) from the United Kingdom with rich (longitudinal) phenotypic and genetic data[28]. Examples of phenotypic data include anthropometric measurements, serum biomarkers, health surveys, death registry linkage, and electronic health record linkage, while genetic data include genome-wide genotyping, imputed data and exome sequencing[19,28]. The UK Biobank resource was approved by the UK Biobank Research Ethics Committee and all participants provided written informed consent to participate. Use of UK Biobank data was performed under application number 17,488 and was approved by the local Massachusetts General Hospital Institutional Review Board.

In the present study, we focused on seven curated disease endpoints defined using self-report, death registry data, and electronic health records (Table II). The assessed curated disease endpoints were atrial fibrillation or flutter (AF), supraventricular tachycardia (SVT), supraventricular tachycardia excluding AF (SVTnoAF), dilated cardiomyopathy (DCM), hypertrophic cardiomyopathy (HCM), ventricular tachycardia (VT) and cardiac conduction system disease (CCD). Baseline and incident events were combined. Baseline characteristics and disease case numbers are presented in Table III and IV.

Exome sequencing was performed on over 454,787 individuals from the UK Biobank as previously described[19, 29]. We utilized the OQFE exome call set and closely followed a previously published pipeline to perform stringent quality control of the exome sequencing data[20]. Briefly, we removed low-quality genotype calls (based on read depth, allele balance and genotype quality), and then removed variants based on call rate (<90%), Hardy-Weinberg equilibrium test (P<1x10-15), presence in low-complexity regions and minor allele count (>0). Sample quality control consisted of the removal of samples with revoked consent, duplicates,

samples with a mismatch between genetically inferred and self-reported sex, samples with low call rates (<90%) and samples that were outliers for several other metrics (transition/transversion ratio, SNV/indel ratio, heterozygous/homozygous ratio, number of singletons). For these metrics, we removed individuals found to be outside of 8 standard deviations from the mean, after regressing out the first 20 ancestral principal components of ancestry. After quality control, we were left with 18,752,405 high-quality autosomal variants and 454,210 high-quality samples, of which 454,162 samples could be matched to their clinical health record data. We further defined a subset of European ancestry determined by an ADMIXTURE[30] algorithm using 1000Genomes[30] as reference (algorithm described previously[32]), consisting of 428,872 participants.

The protein consequences of variants were explored using dbNSFP (v.4.3[33]) and the Loss-of-Function Transcript Effect Estimator (LOFTEE[16]) implemented in the Ensembl Variant Effect Predictor (VEP; v.105[34]) (https://github.com/konradjk/loftee). We focused on variants affecting the Ensembl canonical transcript of *TNNI3K* that were annotated as either missense or as high-confidence loss-of-function (LoF) by LOFTEE. LoF variants flagged by LOFTEE as dubious were removed. Missense variants were assigned a missense score representing the proportion of bioinformatics tools predicting a damaging effect, following previously published methods[20]. In short, we used information from 30 tools included in the dbNSFP database to score each missense variant by the number of tools predicting a damaging/deleterious effect and divided this value by the number of tools that gave a prediction. Missense variants with <7 predictions were removed. For instance, if 14 tools predicted a damaging effect and 28 total tools gave a prediction, then the missense score would equal 0.5 (14/28). Details on the scoring method and included tools have been described in detail previously[20]. We further annotated variants with continental population frequencies from gnomAD (exomes v2[16]) to identify the highest population frequency ("gnomAD POPMAX") among European, South Asian, East Asian, Admixed American, and African continental populations.

We then performed rare variant burden tests to identify associations between rare *TNNI3K* variants and the disease endpoints. To this end, we used a logistic mixed-effects

model using custom software (https://github.com/seanjosephjurgens/UKBB_200KWES_CVD/tree/v1.2), which is a previously-described adaptation of the R-package GENESIS (v2.18[35])[20]. We included as fixed-effects age, age^2, sex, sequencing tranche, ancestral principal components 1 to 4, as well as any principal components from 5 to 20 if associated with the given disease at p<0.05[20]. We included a sparse kinship matrix as a random effect to account for sample relatedness, following the previous methods[29]. Burden tests were run using two-sided Score tests, with p-values computed using the Saddle-Point-Approximation to account for case-control imbalance[36]. Across the disease endpoints, we ran burden testing for 33, potentially correlated, masks constructed using 3 different frequency filters (MAF<0.1%, MAF<0.001%, singletons) and 11 different filters for inclusion of LoF and missense variants utilizing various cutoffs for missense deleteriousness scores (Supplemetary Figure I). Results for tests with cumulative minor allele count (cMAC)<20 were removed to mitigate false-positive signals driven by low allele count. For each phenotype, we then used the Cauchy distribution test[37] to combine the various p-values into a single p-value for the analysis (Supplemetary Figure I). The Cauchy distribution test allows for valid aggregation of multiple, potentially correlated, test statistics into a single omnibus test statistic[37]. The Bonferroni-corrected significance cutoff for the overall analysis was set at 0.00714 (=0.05/7 disease endpoints). Finally, significant results were assessed in a sensitivity analysis restricting to participants of European ancestry only. Odds ratios (ORs) and confidence intervals (CIs) for all masks with MAF<0.1% were estimated using Firth's logistic regression among unrelated samples, adjusting for the same fixed-effects covariates.

cDNA constructs and mutagenesis

The plasmid containing the FLAG-tagged human wild-type (WT) *TNNI3K* cDNA (NM_015978.3; cloned into a pRK5 vector) was provided by Dr. Hao Tang and Prof. Douglas Marchuk (Duke University School of Medicine, Durham, NC). Site-directed mutagenesis was performed with the Quick Change XL kit (Agilent Technologies, Santa Clara, USA) to introduce the *TNNI3K* variants to the WT *TNNI3K* cDNA. The *TNNI3K* variants and their respective mutagenesis primers are summarized in table V.

Cell culture and transfection

HEK293A cells were grown in a 6-well plate in DMEM (21969-035, Gibco, USA) containing 10% FBS (Biowest, France), 1% penicillin-streptomycin (Gibco, USA), and 1% L-glutamine (Gibco, USA) in a 5% CO2 incubator at 37°C. At 70% of confluency, cells were transfected with 2µg WT *TNNI3K* cDNA or *TNNI3K* variant cDNA using 6µL Lipofectamine (Invitrogen, Carlsbad, USA). After 48 hours, protein was extracted using RIPA buffer (0.1% SDS, 1% Triton, 50mM Tris-HCl (pH=8), and 150mM NaCl, 0.5% sodium deoxycholate), supplemented with protease inhibitor cocktail (Roche, CH), and phosphatase inhibitors phenylmethylsulfonyl fluoride (4mM), and sodium orthovanadate (0.5mM, Sigma-Aldrich, USA).

Western blot

Protein samples were heated to T=95°C in 1x Laemmli buffer. Protein samples were loaded onto gradient Mini-PROTEAN TGX Precast gels (4-20%, Biorad, USA). Proteins were transferred onto a PVDF membrane using a semi-dry blotting system (Biorad, USA). The membranes were then blocked for 1 hour using either 3% milk (Protivar, Nutricia, Netherlands) in TBST or BSA (A7888, Sigma, USA) in TBST. TNNI3K protein expression was determined using an anti-FLAG mouse M2 antibody (F3165 (1:500), Sigma, USA) in 3% milk. Auto-phosphorylated TNNI3K protein was detected by anti-phospho-tyrosine mouse antibody PY99 (sc-7020 (1:100), Santa Cruz Biotechnology, USA) in 3% BSA. The loading control GAPDH

was identified with an anti-GAPDH mouse antibody (10R-G109a (1:10,000), Fitzgerald, USA) diluted in 3% milk. Primary antibodies were incubated overnight at T=4°C on a rotator. The membranes were washed three times for 15 minutes with TBST. Then the membranes were subjected to the secondary antibody, anti-mouse horseradish peroxidase-conjugated secondary antibody (NA9310V, GE HealthCare, United Kingdom), for 1h at room temperature. Western blot signal was detected using Amersham ECL Prime Blotting Detection Reagent (GE HealthCare, United Kingdom). Protein expression patterns were visualized by LAS-4000 Lite, Fujifilm. The intensity of the protein bands was analyzed using ImageLab 6.0.1 (BioRad, USA). Phospho-tyrosine and FLAG expression levels were both normalized by GAPDH. Auto-phosphorylation values of TNNI3K variants were normalized to WT TNNI3K.

RNA (splice) analysis

Total RNA was obtained from peripheral blood using PAXgene tubes and the PAXgene blood RNA kit (Qiagen). cDNA was produced by reverse transcription-PCR with the use of Superscript III kit (Invitrogen) and random hexamers. PCRs were performed using *TNNI3K* (NM_015978.3) specific primers located on the transition of exon 15 and 16 (AAACGTTATCGAGCCAATACCT) and in exon 18 (GTGGTGGCAGATTTTGGAG; Merck Sigma-Aldrich). The cDNA was sequenced with the Brilliant Dye Terminator Cycle Sequencing system (Thermofisher Scientific) and Big Dye Terminator kit (Applied Biosystems) and analyzed using CodonCode Aligner version 8.0.2. Splice site predictions were performed according to the algorithms from AlaMut (Sophia Genetics, Lausanne, Switzerland).

Statistical analysis

Enrichment analysis Amsterdam UMC

For the enrichment analysis in the Amsterdam UMC panels, a chi-square test was performed. The threshold for significance was defined as $p<0.008$ (=0.05/6 disease endpoints) corrected for multiple testing with the Bonferroni method.

TNNI3K disease associations in the UK Biobank

For each phenotype, we used the Cauchy distribution test[37] to combine the various p-values into a single p-value for the analysis (Supplemental Figure I). The Cauchy distribution test allows for valid aggregation of multiple, potentially correlated, test statistics into a single omnibus test statistic[37]. The Bonferroni-corrected significance cutoff for the overall analysis was set at 0.00714 (α=0.05/7 disease endpoints). Finally, significant results were assessed in a sensitivity analysis restricted to individuals of European ancestry only.

Auto-phosphorylation assay

Data are expressed as mean ± standard error of the mean (SEM). Comparison of auto-phosphorylation levels that followed normal distributed was performed with one-way repeated measures analysis of variance (ANOVA) with Dunnett's posthoc correction. The Kruskal-Wallis test with Dunn's posthoc correction was used for non-normally distributed data. The threshold for significance was defined as $p<0.05$.

Supplemental Figures and Results

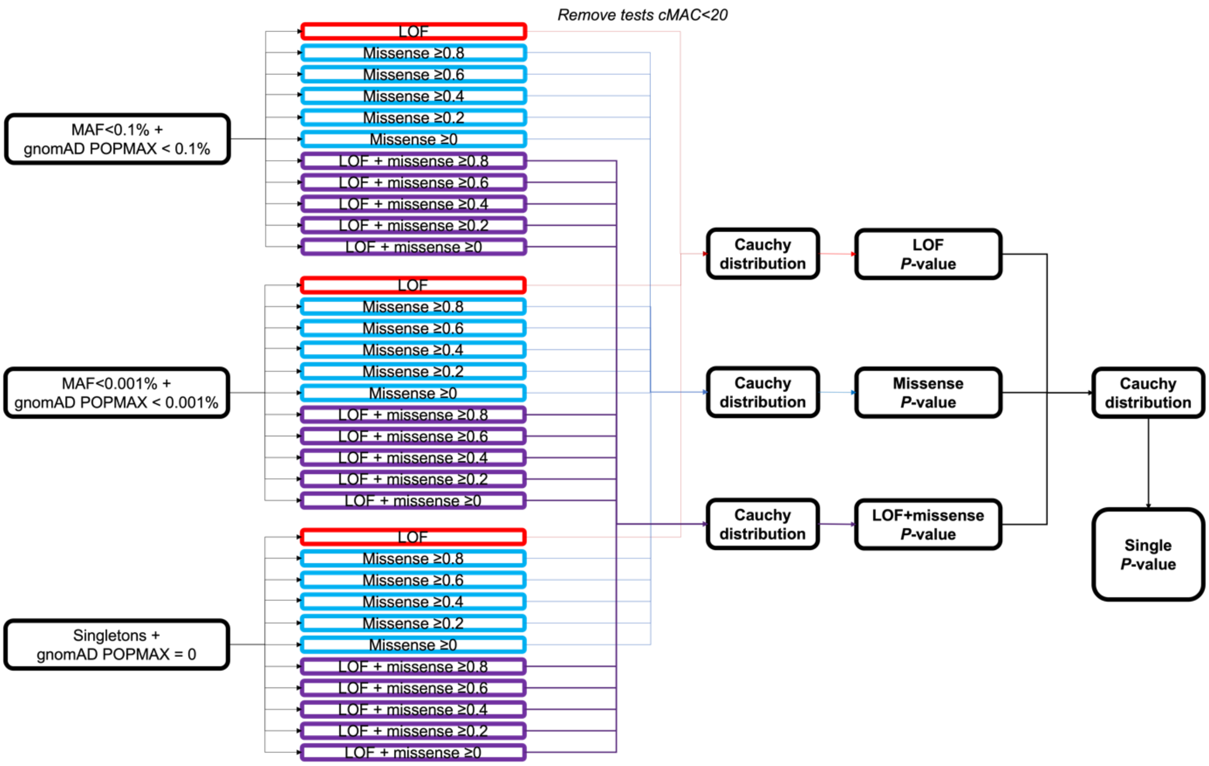

Figure I. Flowchart for gene-based testing of *TNNI3K* rare variants in UK Biobank. As shown on the left, 3 different frequency cutoffs were used (I) MAF<0.1% in the UK Biobank and MAF<0.1% in each of the continental populations in gnomAD, (II) MAF<0.001% in UK Biobank and each continental gnomAD population, and (III) singleton variants in UK Biobank not found in gnomAD continental populations. For each frequency cutoff, we performed a burden test for LOF variants (highlighted in red), 5 tests for missense variants based on different missense score cutoffs (≥0, ≥0.2, ≥0.4, ≥0.6, ≥0.8; highlighted in blue) and 5 tests for LoF variants combined with each of the missense masks (highlighted in purple). Then, p-values for each of the LoF tests were combined into a single p-value using the Cauchy distribution test, as were each of the missense tests and each of the LoF+missense tests. Prior to combining p-values, tests with cMAC<20 were removed. Finally, to produce a single p-value for a given phenotype, we combined the LoF, missense and LoF+missense p-values into a single p-value, again using the Cauchy distribution test.

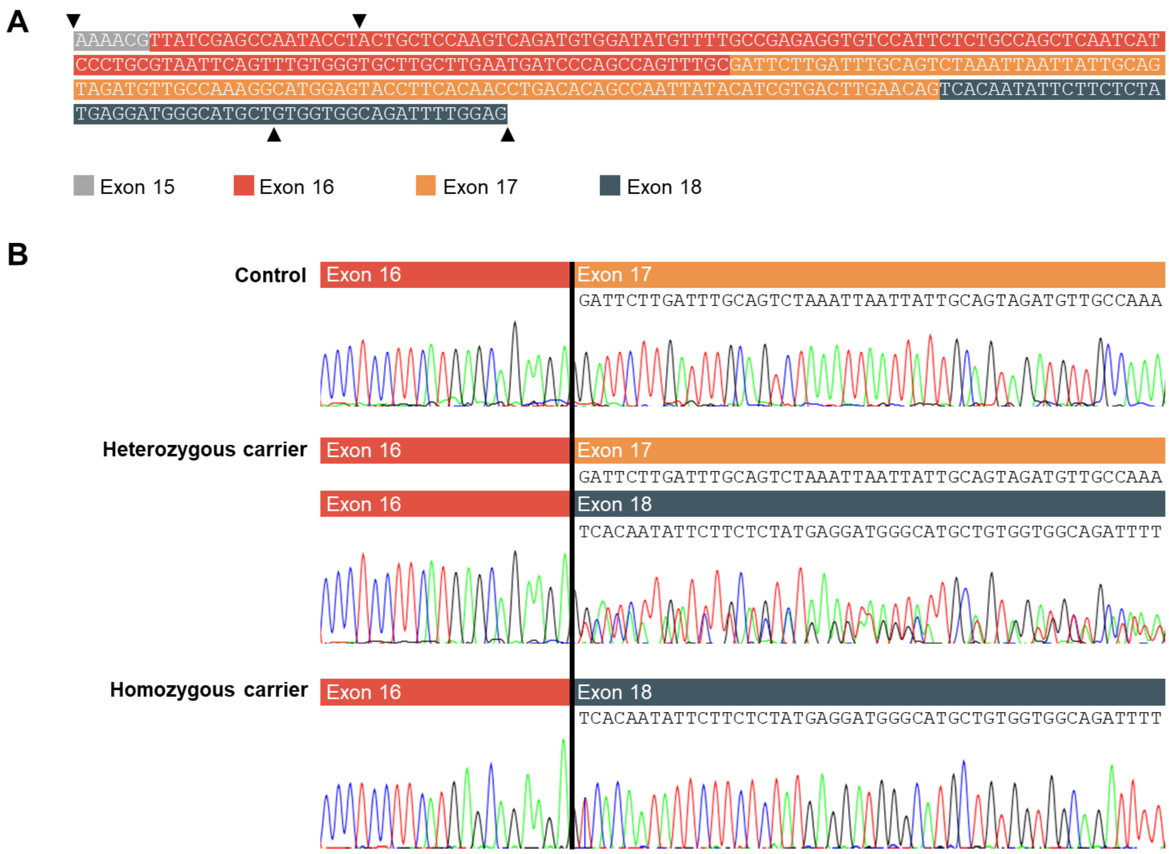

Figure II. Splice analysis of *TNNI3K*-c.1772G>C. A: Part of the *TNNI3K* cDNA sequence. The RT-PCR primers are indicated by the arrowheads. B: Sequence traces of the RT-PCR reactions using peripheral blood from (top to bottom): (I) A control, (II) heterozygous carrier, and (III) homozygous carrier of *TNNI3K*-c.1772G>C. In the heterozygous patient, both the WT transcript (from the normal allele) and the aberrant transcript (missing exon 17) from the r.1772g>c allele are found. In the homozygous patient, only the aberrant transcript missing exon 17 is found.

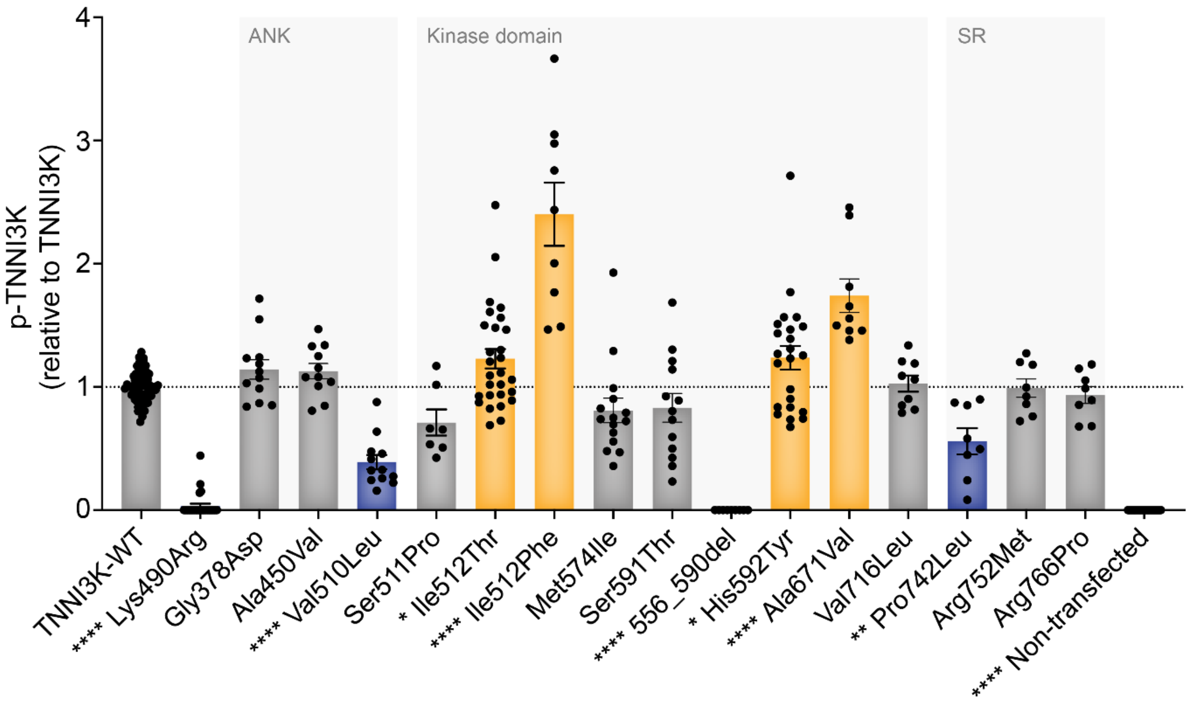

Figure III. Western blot analysis of TNNI3K variants. Each dot represents an independent transfection. p-TNNI3K expression was corrected for FLAG expression. Values are relative to TNNI3K expression. Grey: unchanged auto-phosphorylation. Blue: decreased auto-phosphorylation. Yellow: Increased auto-phosphorylation. ANK: Ankyrin repeat, SR: Serine-rich domain. One-way ANOVA (Dunnett's) and Kruskal-Wallis (Dunn's) vs. TNNI3K *p<0.05, **p<0.01, ***p<0.001, ****p<0.0001 (indicated on the x-axis).

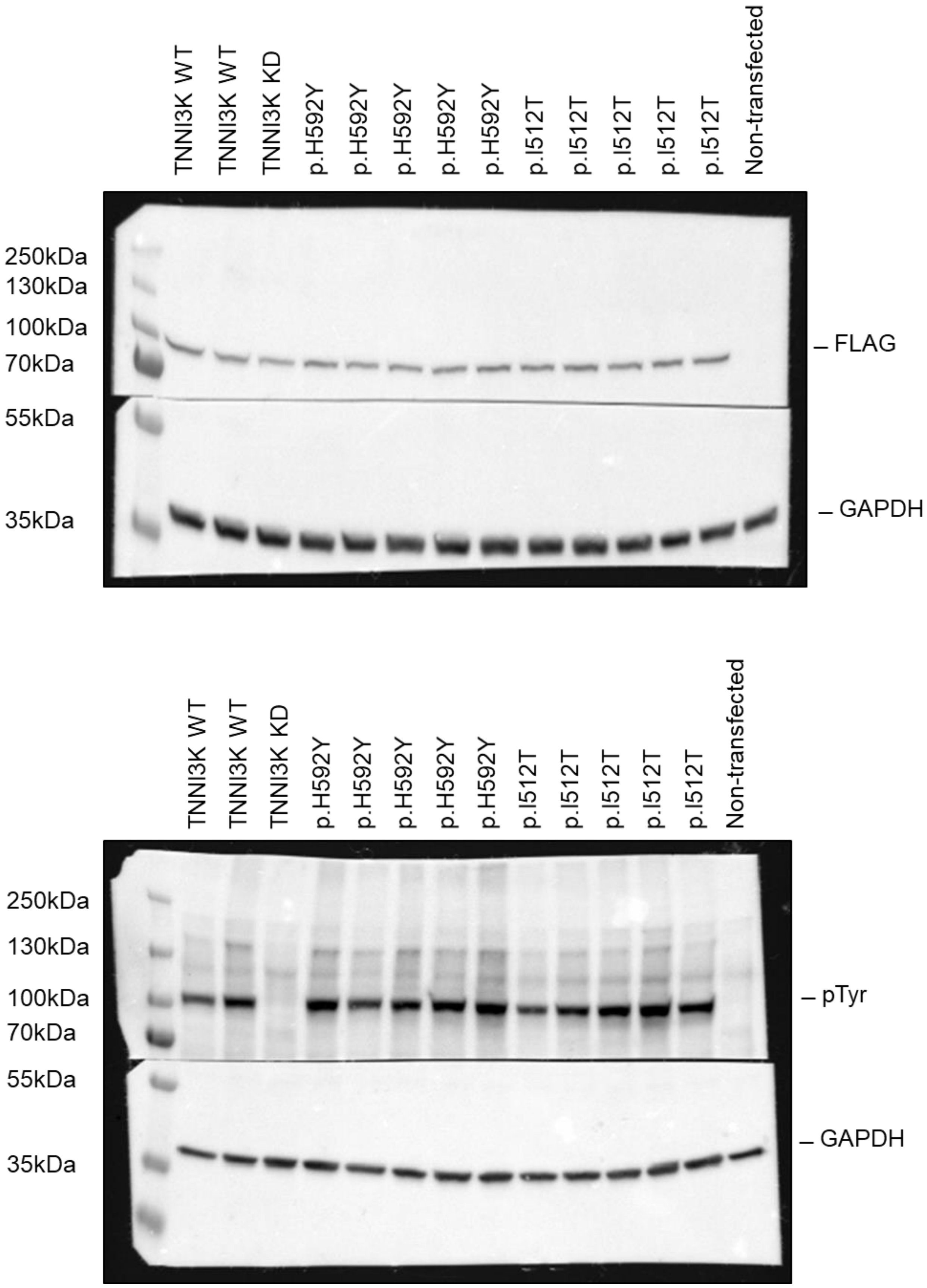

Figure IV. Full Western blot (Figure 4).

TNNI3K-p.Ile512Thr family

The proband (IV-4) is a male carrier of TNNI3K-p.Ile512Thr experienced syncope during exercise at the age of 22. His medical history includes recurrent syncopes while ill. His ECG revealed RBBB and LAD. Echocardiographic examination did not reveal any abnormalities. The ajmaline test was negative. His brother (IV-5) also carried the variant and presented with iRBBB and LAD with no history of syncope or any abnormal echocardiographic findings. Their mother (III-3), carrier of the variant, suffered from syncopes since the age of 14. Her ECG shows LBBB and LAD. Her echocardiographic examination showed borderline systolic function (LVEF=51%) with normal dimensions of the ventricles, first to second-grade tricuspid insufficiency and mild dilatation of the right atrium. Her mother (II-3) was diagnosed with LBBB at the age of 46 and later developed DCM (LVEF=30%) followed by cardiac resynchronization therapy defibrillator (CRT-D) implantation. She also tested positive for the variant. Family history was also positive for multiple cases of AF and SCD at a young age. Both the proband (IV-4) and the mother (III-3) also carried an additional class 3 variant in *LAMA4* (*LAMA4* c.2555C>T; p.(Thr852Met)) that did not co-segregate with the phenotype.

TNNI3K-p.His592Tyr families

Family 1

The proband is a 69-year-old man (1.II-4), who carries TNNI3K-p.His592Tyr. At the age of 51, the patient was admitted to the Cardiology Department with dyspnea and diagnosed with DCM, severe mitral insufficiency (MI), and a left ventricular ejection fraction (LVEF) of 20%. His first 12-lead ECG at admission revealed 1st grade AV block (PQ-interval=220ms), LBBB, LAD, and abnormal ST-segments. He underwent a pacemaker implantation two years later and his LVEF increased over time to 35% with an improvement of mitral valve function (MI from 3rd to 2nd grade). Further genetic screening of this family detected the TNNI3K-p.His592Tyr in both daughters of the proband. Anamnestic indication of further cardiomyopathy in the family had no follow-up due to lack of informed consent.

The 48-year-old daughter (1.III-2), who was found to be a carrier of the variant, presented with mild LV dysfunction with round morphology of LV at the age of 32. Her echocardiographic examination at admission showed normal dimensions and decreased function of the LV. Magnetic resonance imaging (MRI) of the heart revealed decreased LVEF (50-63%). Over the years, heart function and morphology did not worsen. Her latest echocardiographic examination showed round LV morphology with LVEF=45%. Her treatment included candesartan. She suffers from palpitations and fatigability.

The son of the proband (1.III-3) demonstrated no symptoms at age of 46. His echocardiographic examination showed LVEF=46% with normal dimensions of all cardiac chambers, normal diastolic function, and LVEDD=49mm. His ECG and Holter examination did not reveal any conduction abnormalities or arrhythmias. He is not a carrier of the variant.

The younger daughter of the proband (1.III-4) presented with mild DCM with borderline dimensions, round morphology of LV, and decreased function of LV at the age of 33. MRI confirmed these findings (LVEF=46% and LVEDD=55mm), showing no signs of fibrosis. 12-lead ECG, Holter examination, and a stress test did not detect any abnormalities. She also carries the TNNI3K-p.His592Tyr variant.

Family 2

The 68-year-old proband (2.II-1) experienced out-of-hospital cardiac arrest (OHCA) and was diagnosed with DCM at the age of 54. Echocardiography and MRI showed dyssynchrony and reduced LVEF equal to 25% and 43%, respectively. Her treatment included an implantable cardioverter-defibrillator (ICD) implantation. ECG recordings revealed LBBB and borderline first-degree AV block (PR=200ms).

Her mother (2.I-3) was diagnosed with a third-grade AV block at the age of 65, which was followed by pacemaker implantation. The TNNI3K variant His592Tyr was also detected in her.

Family 3

The proband is a 33-year-old woman (3.II-1) with a history of broad complex tachycardia (most probably AVNRT with aberrant conduction) during the exercise test at the age of 27. A year later she developed atrial flutter and atypical AVNRT, for which she underwent ablation. Her exercise test showed broad complex tachycardia with LBBB morphology (suspected non-sustained ventricular tachycardia (nVT)) with no abnormalities in MRI and echocardiographic examination. After re-ablation her ECG revealed a ventricular escape rhythm. Holter examination revealed frequent symptomatic PVCs (palpitations during PVCs). Her medical history also included AV block and well-documented vasovagal syncope. Her latest examinations did not reveal any arrhythmias or morphological abnormalities. She also experienced syncope, which was evaluated to be of vasovagal origin. Her mother (3.I-2) tested negative for the *TNNI3K* variant and did not demonstrate any cardiac arrhythmias or cardiomyopathy. She did have an aortic valve insufficiency.

Family 4

The proband is a 52-year-old woman (4.II-2) with a diagnosis of peripartum cardiomyopathy with hemodynamic decompensation (LVEF=28%) at the age of 34. LV function recovered partially with dilatation of the LV and LA and sinus tachycardia at rest. Seven years later she demonstrated recurrent AVNRT at exercise. At the age of 47, she underwent an unsuccessful ablation of a slow pathway, followed by possible junctional tachycardia. Her Holter examination was associated palpitations with episodes of junctional tachycardia. A year later the ablation procedure was repeated and the patient did not present any symptoms of SVTs. The 12-lead ECG did not reveal any abnormalities within 3 months after ablation. Additionally to the *TNNI3K* variant, she is a carrier of a variant in *RBM20*-c.3115C>T p.(Pro1039Ser), which is of unknown significance. The first daughter of the proband (4.III-I), tested positive for the TNNI3K variant, her clinical examination did not reveal any abnormalities. Her younger sister tested negative for the variant and is healthy (4.III-2).

Family 5

The proband is a 68-year-old woman (5.II-2) with a long history of DCM and recurrent arrhythmias. Since the age of 58, she experienced syncope during episodes of atrial tachycardias, for which she underwent a partially successful ablation. Her echocardiographic examination revealed mild diastolic dysfunction. Six years later she started experiencing broad complex tachycardia episodes (Sustained VT with LBBB aberrant conduction). Her history also includes paroxysmal AF with spontaneous conversion and frequent PVCs. After the second catheter ablation, she did not suffer from palpitations. The latest MRI showed LV dilation, LVEF=34%, and LVEED=62mm. The latest echocardiographic examination suggested a mild concentric remodeling with normal dimensions of the heart chambers. One of the daughters (5.III-2) was shown to carry the variant. Cardiac examination (at age 41) did not demonstrate any cardiac arrhythmias or cardiomyopathy.

Family 6

The proband (6.V-1) carries the TNNI3K-p.His592Tyr variant and died suddenly at the age of 56. He was diagnosed with DCM and AF at the age of 52. His brother (6.V-2) also carries the variant and showed VT during an exercise test and underwent ICD implantation. His sister (6.V-3) has a history of AF episodes, her genetic status is unknown. Their mother (6.IV-2) is also a carrier of the variant in *TNNI3K* and experienced cardiac arrhythmias (palpitations) at the age of 43. Later, she developed paroxysmal AF, nVTs, and LBBB. She also underwent a ICD implantation. Her mother (6.III-1) was diagnosed with arrhythmias at age 37 and died from SCD at age 41. The family history further includes multiple cases of SCD at a young age (see figure 3F).
